# Dynamics and Functional Interplay of Nonhistone Lysine Crotonylome and Ubiquitylome in Vascular Smooth Muscle Cell Phenotypic Remodeling

**DOI:** 10.3389/fcvm.2022.783739

**Published:** 2022-03-08

**Authors:** Shan-Hu Cao, Zhi-Huan Chen, Ru-Yuan Ma, Lin Yue, Han-Mei Jiang, Li-Hua Dong

**Affiliations:** Key Laboratory of Medical Biotechnology of Hebei Province, Key Laboratory of Neural and Vascular Biology of Ministry of Education, Cardiovascular Medical Science Center, Department of Biochemistry and Molecular Biology, College of Basic Medicine, Hebei Medical University, Shijiazhuang, China

**Keywords:** vascular smooth muscle cells, phenotypic remodeling, lysine crotonylation, lysine ubiquitylation, PPI analysis

## Abstract

**Background:**

The crotonylation of histones is discovered of late as one of the post-translational modifications (PTMs) that can regulate gene expression. However, the function of crotonylation on nonhistone proteins in vascular smooth muscle cells (VSMCs) is unclear. Here, we aim to find the cellular characteristics of crotonylated nonhistone proteins and the cross talk with ubiquitinated proteins in VSMC phenotypic remodeling using the modified omics and proteomic analysis.

**Methods:**

We performed the modified omics and proteomic analysis of VSMCs before and after the stimulation with platelet-derived growth factor-BB (PDGF-BB). The crotonylated and ubiquitinated pan-antibody was used to enrich proteins and then subjected to a high-throughput mass spectrometry analysis. The enrichment analysis was performed within differentially modified proteins in regard to GO terms, KEGG, and protein domains.

**Results:**

As a result, there were 2,138 crotonylation sites in 534 proteins and 1,359 ubiquitination sites corresponding to 657 proteins. These crotonylated proteins detected after PDGF-BB stimulation might be involved in various vital cellular pathways and carry out important functions in VSMCs. Some of them closely took part in significant physiological processes of VSMC phenotypic remodeling, including glycolysis/gluconeogenesis, vascular smooth muscle contraction, and the PI3K-Akt signaling pathway. Furthermore, the KEGG pathway enrichment analysis showed the involvement of ubiquitinated proteins in the physiological processes of VSMC phenotypic remodeling, including glycolysis/gluconeogenesis, vascular smooth muscle contraction, RAS signaling pathway, or the PI3K-Akt signaling pathway. A cross talk analysis showed that there were 199 sites within the 177 proteins modified by crotonylation and ubiquitination simultaneously. Protein–protein interaction (PPI) network analysis indicated that crotonylated and ubiquitinated proteins play an important role in cellular bioprocess commonly and possibly have a synergistic effect.

**Conclusion:**

In summary, our bioinformatics analysis shows that the crotonylation and ubiquitination of nonhistone proteins play an essential role in VSMC phenotypic transformation induced by PDGF-BB stimulation. The cross talk between crotonylation and ubiquitination in glycolysis is possibly a novel mechanism during VSMC phenotypic remodeling.

## Introduction

Cardiovascular diseases remain the number one primary cause of death for patients all over the world, and the risk factors of cardiovascular disease are gradually increasing among young people (1). Vascular smooth muscle cells (VSMCs) carry out phenotypic switching when blood vessels are injured, and phenotypic transformation is the first step of the pathological basis of vascular diseases (2). Phenotypic transformation of smooth muscle cells leads to vascular remodeling when vascular diseases such as atherosclerosis, diabetic macroangiopathy, and restenosis occur (3, 4).

After vascular injury, the serum with growth factors, cytokines, and other components in contact with smooth muscle cells activates complex signaling pathways and cellular events. Among cellular activation events, numerous studies have focused on growth factors, which could contribute to the formation of atherosclerotic and restenosis lesions (5, 6). We focus our study exclusively on the role of platelet-derived growth factor-BB (PDGF-BB) whose function is a chemoattractant or mitogen for VSMC (7, 8). When the blood vessel is enduring injury, PDGF-BB could lead to phenotypic modulation, and the cellular gene expression involved in pattern changing is upregulated (9).

Post-translational modifications (PTMs) have been identified as the most foundational and complicated mechanisms that can regulate various cellular events, including genetic expression, protein biosynthesis, metabolism, and cell cycling (10–12). An extensive interplay between the kinase pathways that regulate VSMC phenotypic remodeling is heavily regulated by several PTMs. In our previous study, tumor necrosis factor receptor-associated factor 6- (TRAF6-) induced SM22α ubiquitination could result in the maintenance of survival advantage to VSMCs by increasing the activity and production of G6PD (13). Recently, we found that TNF-α concurrently caused SIRT1 phosphorylation by virtue of CKII and protected VSMC from inflammation (14). Phosphorylation of SIRT1 makes it interact with and deacetylate EZH2 and, thereafter, promote SM22α transcription *via* EZH2 activity suppression (14). PTMs mainly occur at the lysine residue on account of their specific spatial structure. In addition, among several well-studied types of lysine acetylation (15), malonylation (16), ubiquitination (17), and methylation (18), lysine crotonylation (Kcr) is a newly identified and proven type of PTM in which a crotonyl group is conveyed to a lateral chain of lysine.

Initially, Ten et al. (19) found that lysine crotonylation was identified on histone proteins, which had 28 crotonylated lysine sites. These crotonylated lysine sites of histone hold the crotonyl on ε-amino, same as histone acetylation. Researchers found that the distribution of crotonylation mostly focuses on the transcription starting sites and enhancers of active chromatin, resulting in the change of chromatin structures and frequent occurrence of histone replacement (19). Lysine crotonylation takes precedence over acetylation to label “escape genes,” in course of gender inactivation after meiosis in mouse testes (20). The overlap sites of lysine crotonylation and acetylation are both catalyzed by acetyltransferase named p300/CBP (21). Then, several regulatory enzymes, such as histone acetyltransferase (HAT) p300 (21), HDAC3 (22), and SIRT1/2/3 (23–26), for histone crotonylation have been described.

Recently, the upgrade of mass spectrometry and the enrichment methods of crotonyl peptides have been promoting the identification of crotonylated sites in microorganisms and mammals. The analysis of crotonylome has been reported in HEK293 cell (27) and mouse liver (28) and the maintenance of patients with hemodialysis (29). However, there are still relatively few studies on the crotonylation of nonhistone proteins. Recently, Xu et al. described nonhistone crotonylation in the first place. They showed that some enzymes, which mediate acetylation/deacetylation, could intervene during nonhistone protein crotonylation/decrotonylation. These nonhistone proteins may perform varied cell biological functions and take part in many signaling pathways. This significant finding could inspire researchers to explore nonhistone crotonylation and to elucidate more protein functions upon precise regulation (30).

Ubiquitylation is one of the PTMs, in which ubiquitin holds a covalent binding with the target proteins on lysine. When this modification appears on the proteins, the proteasome and lysosomes will be recruited for degradation and the protein localization will be altered similar to protein function, transport, and interaction. For example, yes kinase-associated proteins (YAPs) could be ubiquitinated by TRAF6 at K252. This contributes to the reducing interaction with angiomotin and more YAPs relocating toward the nucleus (31). Ubiquitin-mediated signaling is frequently altered in cancer (32). Chu et al. provided evidence that the linear ubiquitination could happen on ATG13 and affected its stabilization, resulting in the inhibition of autophagy and maturation of autophagosomes, which are mediated by linear ubiquitin chain assembly complex (LUBAC), E3 ubiquitin ligase complex, and a deubiquitinating enzyme (DUB) OTULIN cooperatively (33). Up to now, the underlying role of ubiquitylation remains unclear during VSMC phenotypic remodeling, and a cross talk between various modifications still needs to be explored in the cardiovascular system.

In this paper, we combined the applications of several technologies, including stable isotope labeling by amino acids in cell culture (SILAC), enrichment affinity using antibodies, along with high-resolution liquid chromatography-tandem mass spectrometry (LC-MS/MS) to compare the quantitative analysis of the crotonylation and ubiquitylation of VSMCs before and after PDGF-BB treatment. So far, there is no relevant research on crotonylation and ubiquitination and their cross talk analysis in cardiovascular diseases. This whole proteome and modified omics comparison could facilitate understanding the fundamental pathological process of vascular diseases related to VSMC phenotypic remodeling.

## Materials and Methods

### Preparation of VSMC Extraction for LC-MS

Vascular smooth muscle cells were isolated from the thoracic aorta of 80–100 g male Sprague–Dawley rats. VSMCs were grown in low glucose Dulbecco's modified Eagle's medium (DMEM) (Invitrogen, USA) with 10% fetal bovine serum (FBS), 100 U/ml penicillin, and 100 μg/ml streptomycin. VSMCs were maintained at 37°C in a humidified atmosphere containing 5% CO_2_, and only passages 3–5 cells at 70–80% confluence were used in the experiments, except if stated otherwise. HEK293 cells were cultured in high glucose DMEM containing 10% FBS. This study was performed *via* a protocol approved by the Institutional Animal Care and Use Committee of Hebei Medical University, in accordance with the Guide for the Care and Use of Laboratory Animals, and the Hebei Medical University Clinical Research Ethics Committee.

Vascular smooth muscle cells with or without PDGF-BB stimulation were collected and treated three times by sonication on ice with a high intensity ultrasonic processor (Scientz) in this kind of lysis buffer (3 μM TSA, 50 mM NAM, 8 M urea, and 1% Protease Inhibitor Cocktail). The supernatant was transferred to new tubes after centrifugation at 12,000 *g* at 4°C for 10 min. Finally, the protein concentration was detected with a BCA kit based on the manufacturer's instructions.

### Trypsin Digestion

For digestion, dithiothreitol (5 mM) was used to reduce the total proteins, lasting for 30 min at the temperature of 56°C, and iodoacetamide (11 mM) was used to alkylate at room temperature lasting for 15 min in darkness. NH_4_HCO_3_ (100 mM) was added to the concentration of 2M. Finally, trypsin was added with the ratio of 1:50 overnight and 1:100 ratio for 4 h.

### High-Performance Liquid Chromatography Fractionation

High pH reverse-phase high-performance liquid chromatography (HPLC) was used to fractionate the tryptic peptides into fractions by using a Thermo Betasil C18 column (5 μm particles, 10 mm ID, and 250 mm length). Briefly, peptides were distributed in a gradient of 8–32% acetonitrile (pH 9.0) lasting for 60 min and then clustered into 60 fractions. At last, 10 fractions appeared, and they were dried by vacuum centrifuging.

### Affinity Enrichment of Crotonylated Peptides

The approach of enrichment of peptides was as follows. NETN buffer was used to dissolve the peptides, and prewashed beads were added to shake at 4°C overnight. After washing by NETN and H_2_O four times and two times, respectively, 0.1% trifluoroacetic acid was used to elute the beads. Finally, the fractions were dried at vacuum for LC-MS/MS.

### LC-MS/MS Analysis

The peptides were loaded onto a reversed phase analytical column (15-cm length and 75 μm i.d.) after dissolving with 0.1% formic acid (solvent A). Based on an EASY-nLC 1,000 UPLC system, at a constant flow rate of 400 nl/min, the gradient was distributed from 6 to 23% solvent B (0.1% formic acid in 98% acetonitrile), lasting for 26 min, 23 to 35% for 8 min, and up to 80% for 3 min. NSI source was used to analyze the peptide after combining tandem mass spectrometry (MS/MS) in Q ExactiveTM Plus (Thermo) with online to the UPLC. The detailed treatment parameters are provided in Supplementary Materials.

### Database Search

Based on the Maxquant search engine (v.1.5.2.8), MS/MS data could be processed. *UniProt* database was searched with reverse decoy database. The STRING database was used to search for the PPI. Other descriptions of the parameters or standards could be seen in Supplementary Materials.

### Statistical Analysis

The quantification of proteomic data was processed by Maxquant software (version 1.5.2.8). It should be noted that the common peptides shared by different groups were excluded from quantification. The *t*-test was used to analyze the significant differences between replicates. We took the specific fold change >1.2 and the value of *p* < 0.05 of proteins into account to perform differential analysis. The other data were analyzed with a one-way ANOVA using SPSS software. *p* ≤ 0.05 was thought to be statistically significant.

## Results

### Quantitative Comparison of the Crotonylome of VSMC Phenotypic Transformation

**Figure 1:**
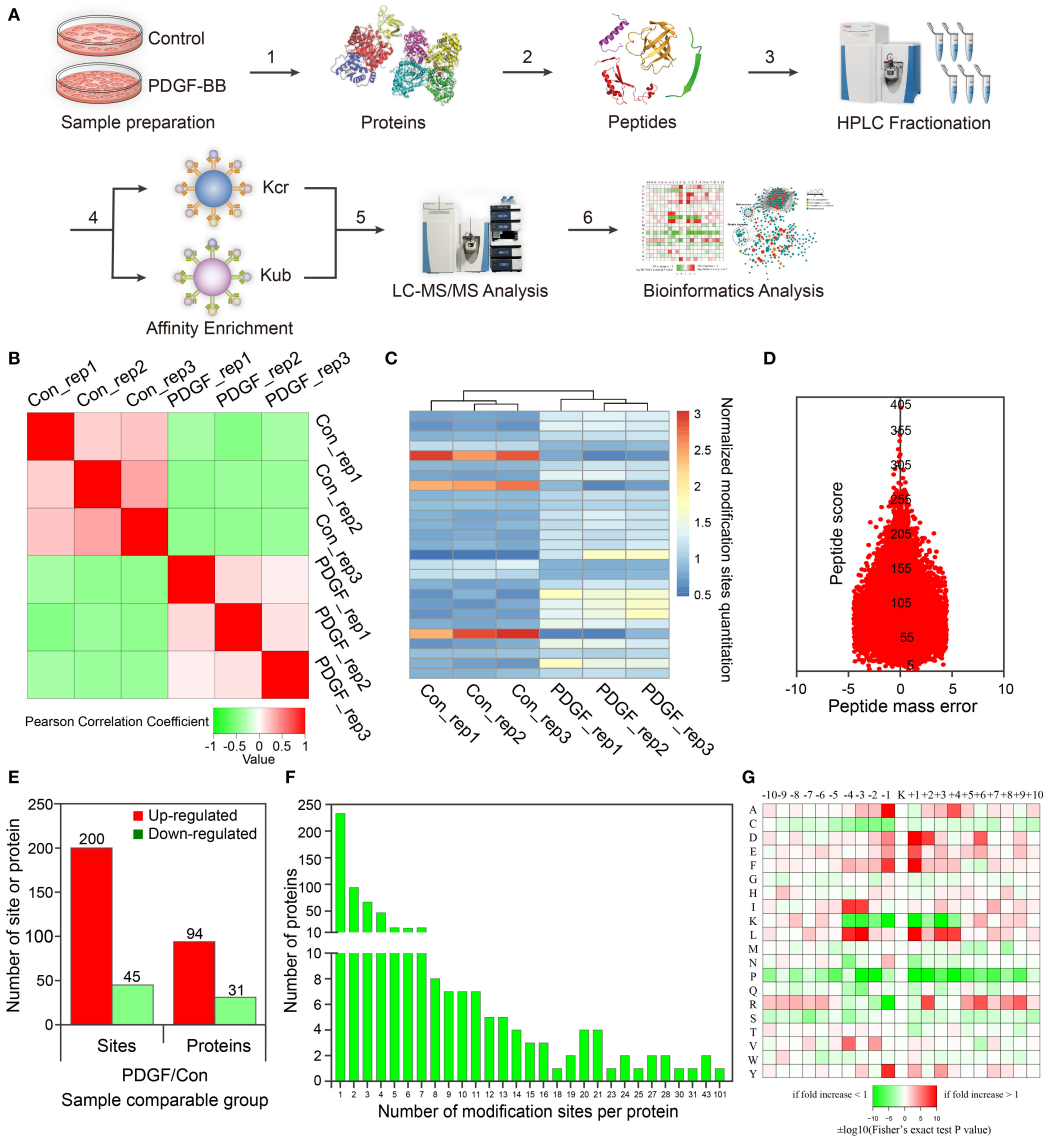
Proteomic and crotonylomic profiling showed significant differences in vascular smooth muscle cells (VSMCs) upon platelet-derived growth factor-BB (PDGF-BB) treatment and control group. **(A)** A systematic workflow for quantitative profiling of global crotonylome. VSMCs pre- and post-treatment with PDGF-BB were extracted (step 1) and digested with trypsin enzyme (step 2). High-performance liquid chromatography (HPLC) was used to separate the peptides (step 3), and we then analyzed the peptides with mass spectrometry (MS) (step 5) after affinity enrichment using the pan anti-crotonyl-lysine monoclonal antibody and the pan anti-ubiquitinate-lysine monoclonal antibody (step 4). Finally, we use the data from MS for bioinformatics analysis (step 6). **(B)** A heat map was designed to calculate Pearson's correlation coefficient using pairings of all samples. This coefficient is a value to measure the degree of linear correlation between the two sets of data: the closer Pearson coefficient is to −1, the negative correlation is observed; the closer Pearson coefficient is to 1, the positive correlation is observed; the closer Pearson coefficient is to 0, the unrelated correlation is observed. Red shows a correlation coefficient of 1, green shows a correlation coefficient of −1, and white shows a correlation coefficient of 0. **(C)** The heat map shows the quantitative differences in different modification sites between the two groups. **(D)** The mass error of the whole identified peptides. **(E)** Differentially expressed proteins and modification sites in the PDGF group relative to the Con group. **(F)** Statistical analysis of lysine crotonylation sites in all proteins. **(G)** Motif enrichment heat map of all the upstream and downstream amino acids of the identified crotonylation modification sites. Con represents the group of control without PDGF-BB treatment, and PDGF represents the group with PDGF-BB treatment. Rep represents repeat sample.

It is demonstrated that PDGF-BB could regulate VSMC differentiation at the concentration of 10 ng/ml (34). Then here, VSMCs were incubated with PDGF-BB (10 ng/ml) for 1, 3, 6, 12, 24, 48, and 72 h. We examined the changes of several PTMs during the phenotypic transformation of VSMC. As shown in Supplementary Figure S1A, the expression of the smooth muscle contractile proteins SM α-actin and SM22α decreased at 24 h, which indicated a VSMC transformation toward a proliferative phenotype at 24 h after PDGF-BB stimulation. Moreover, the pan-crotonylated level increased at 3 h after PDGF-BB stimulation and peak at 24 h (Supplementary Figure S1B). For other types of PTMs, with the prolonged time of PDGF-BB stimulation, there was no consistency in the level of PTM and the phenotypic transformation (Supplementary Figures S1C–H). Subsequently, the proteins derived from VSMCs before and after PDGF-BB treatment were separately identified by LC-MS/MS-based quantitative analysis within three random repetitions according to the experimental flowchart (Figure 1A).

For these repetitions, we tested the quantitative statistical consistency of the biological or technical replicates. The heat map shows an obvious positive correlation between sample replicates and a negative correlation between different treatment groups (Figure 1B). Another quantitation heat map shows that there is a high similarity between the duplicated samples but modification sites have changed significantly after PDGF-BB treatment (Figure 1C). To confirm the MS data, the mass error was used to evaluate the accuracy of the MS data of the detected peptides. The mass errors were concentrated in the vicinity of 0 and most of them were <0.02 Da, proving that the MS data were relatively accurate (Figure 1D).

In this work, 2,386 crotonylation sites corresponding to 570 proteins and 2,138 crotonylation sites from 534 proteins were quantified in VSMCs with or without PDGF-BB treatment (Table 1). Among the 534 quantifiable proteins, 245 crotonylation sites from 125 proteins changed over 1.3-fold (200 sites from 94 proteins upregulated and 45 sites from 31 proteins downregulated) (Figure 1E). We also performed statistics on the proteins and sites corresponding to other fold differences (Table 2). Of these 570 crotonylated proteins, 233 (40.9%) have only one crotonylation site, 94 (16.5%) contain two sites, and 67 (11.8%) possess three. In total, 58 (10.2%) proteins get 10 more crotonylation sites (Figure 1F), such as pyruvate kinase (PK), which contains 12 crotonylated sites (Table 3).

**Table 1:** Tandem mass spectrometry (MS/MS) spectrum database search analysis summary of crotonyl-omics.

**Identified proteins**	**Quantifiable proteins**	**Identified sites**	**Quantifiable sites**
570	534	2,368	2,138

**Table 2:** Differentially changed modification sites (modified proteins) summary of crotonyl-omics (filtered with the threshold value of expression fold change).

**Compare group**	**Regulated type**	**Fold change > 1.2**	**Fold change > 1.3**	**Fold change > 1.5**	**Fold change > 2**
PDGF/Con	Upregulated	241 (107)	200 (94)	100 (58)	14 (9)
	Downregulated	49 (35)	45 (31)	31 (20)	14 (7)

**Table 3:** Modification site information of pyruvate kinase.

**Position**	**Amino acid**	**Protein description**	**Score**	**Modified sequence**	**Mass error (ppm)**	**MS/MS Count**
269	K	Pyruvate kinase	104.05	VFLAQK(1)MMIGR	1.4519	12
261	K	Pyruvate kinase	91.906	VLGEK(1)GK	−0.41152	5
305	K	Pyruvate kinase	116.58	GDLGIEIPAEK(1)VFLAQK	0.27956	23
115	K	Pyruvate kinase	151.91	PVAVALDTK(1)GPEIR	−1.5825	15
367	K	Pyruvate kinase	112.26	IMLSGETAK(1)GDYPLEAVR	0.53367	8
498	K	Pyruvate kinase	109.15	VNLAMNVGK(1)AR	2.312	11
186	K	Pyruvate kinase	97.823	IYVDDGLISLQVK(1)EK	2.1065	10
89	K	Pyruvate kinase	124.84	THEYHAETIK(1)NVR	1.6963	14
224	K	Pyruvate kinase	137.72	LPAVSEK(1)DIQDLK	0.39533	19
62	K	Pyruvate kinase	94.692	SVEMLK(1)EMIK	0.82137	30
311	K	Pyruvate kinase	104.05	VFLAQK(1)MMIGR	1.4519	12
270	K	Pyruvate kinase	143.97	IISK(1)IENHEGVR	0.5539	9

To identify the bias of amino acids adjacent to the crotonylation lysine sites, we observed both sides of the crotonylation sites, from −10 to +10 positions of lysine. This analysis showed that there were a total of 2,731 peptides containing crotonylation sites. The motifs adjacent to crotonylation sites showed a characterization of distinct patterns (Table 4). Among them, the peptides included 10 specific and conserved motifs, such as ..........KcrL.......... (368 peptides), ..........KcrE.......... (334 peptides), and ..........EKcr.......... (331 peptides) (Supplementary Figure S2). Motif enrichment heat map showed that aspartic acid (D) and glutamic acid (E) residues were overenriched at the −1 and +1 positions beside the crotonylation sites (Figure 1G). The distribution of other residues is as follows: alanine (A) is at −1/+4 positions, phenylalanine (F) is at −1/+1, isoleucine (I), and leucine (L) are at −3/−4, arginine (R) is at +2, and tyrosine (Y) is at −1. Crotonylation neutralizes the positive charge of lysine and might weaken its interaction with surrounding negatively charged amino acids, such as E and D. These data showed that numerous nonhistone proteins are modified by crotonylation in VSMCs for the first time.

**Table 4:** The characteristic sequence of the modified site obtained by motif analysis.

**Motif**	**Motif score**	**Foreground**		**Background**		**Fold increase**
		**Matches**	**Size**	**Matches**	**Size**	
.........AK..........	16	247	2,270	35,327	601,417	1.85
..........KF.........	16	149	2,023	17,268	566,090	2.41
..........KL.........	14.75	277	1,874	49,987	548,822	1.62
..........KD.........	16	190	1,597	31,385	498,835	1.89
..........KE.........	12.92	238	1,407	48,851	467,450	1.62
..........K.D........	9.94	100	1,169	17,939	418,599	2
........A.K..........	9.62	121	1,069	24,850	400,660	1.82
.........EK..........	6.66	137	948	34,965	375,810	1.55
.........FK..........	6.62	60	811	12,313	340,845	2.05
.........YK..........	6.49	52	751	10,534	328,532	2.16

### Enrichment Analyses of Lysine Crotonylated Proteins, Cluster Analysis of Functions of Them With Different Fold Changes, and STRING Interaction Network of Crotonylated Proteins With Cytoscape

To explore the significance of crotonylation, we classified crotonylated proteins from the following aspects, including subcellular localization, cellular component, biological processes, and molecular function. In the upregulated crotonylated proteins, we figured out the distribution of them, 46% in the cytoplasm, 21% in the nucleus, and 13% in the mitochondria (Supplementary Figure S3A). On the contrary, in the downregulated crotonylated proteins, there was a similar result, 32% in the cytoplasm, 26% in the nucleus, and 13% in the mitochondria (Supplementary Figure S3A). The classification of a cellular component of crotonylated proteins was done using the gene ontology annotation (Supplementary Figure S3B). The upregulated crotonylated proteins were widely distributed in the organelles (22%), the cell (22%), and the macromolecular complexes (14%). However, the downregulated ones were distributed in the organelles (23%), the cell (23%), and membranes (16%).

The analysis of the biological process involved in crotonylated proteins showed that, among the upregulated proteins, 18% were in cellular processes, 13% were in each single-organism process and metabolic process, respectively. On the contrary, the distribution of processes involving the downregulated crotonylated proteins was as follows: 17% in cellular processes, 16% in the single-organism process, and 13% in biological regulation (Supplementary Figure S3B). The molecular function of these crotonylated proteins was assigned to several groups based on the GO annotation. In the upregulated crotonylated proteins, 53% were involved in binding, 19% were related to structural molecule activity, and 18% were associated with catalytic activity. In the downregulated crotonylated proteins, 60% were associated with binding, 15 and 9% were related to catalytic and structural molecule activities, respectively (Supplementary Figure S3B). These results indicated that there is a difference in upregulated and downregulated crotonylated proteins, which are so widely separated or spread in subcellular localization, cellular components, biological processes, and molecular function.

**Figure 2:**
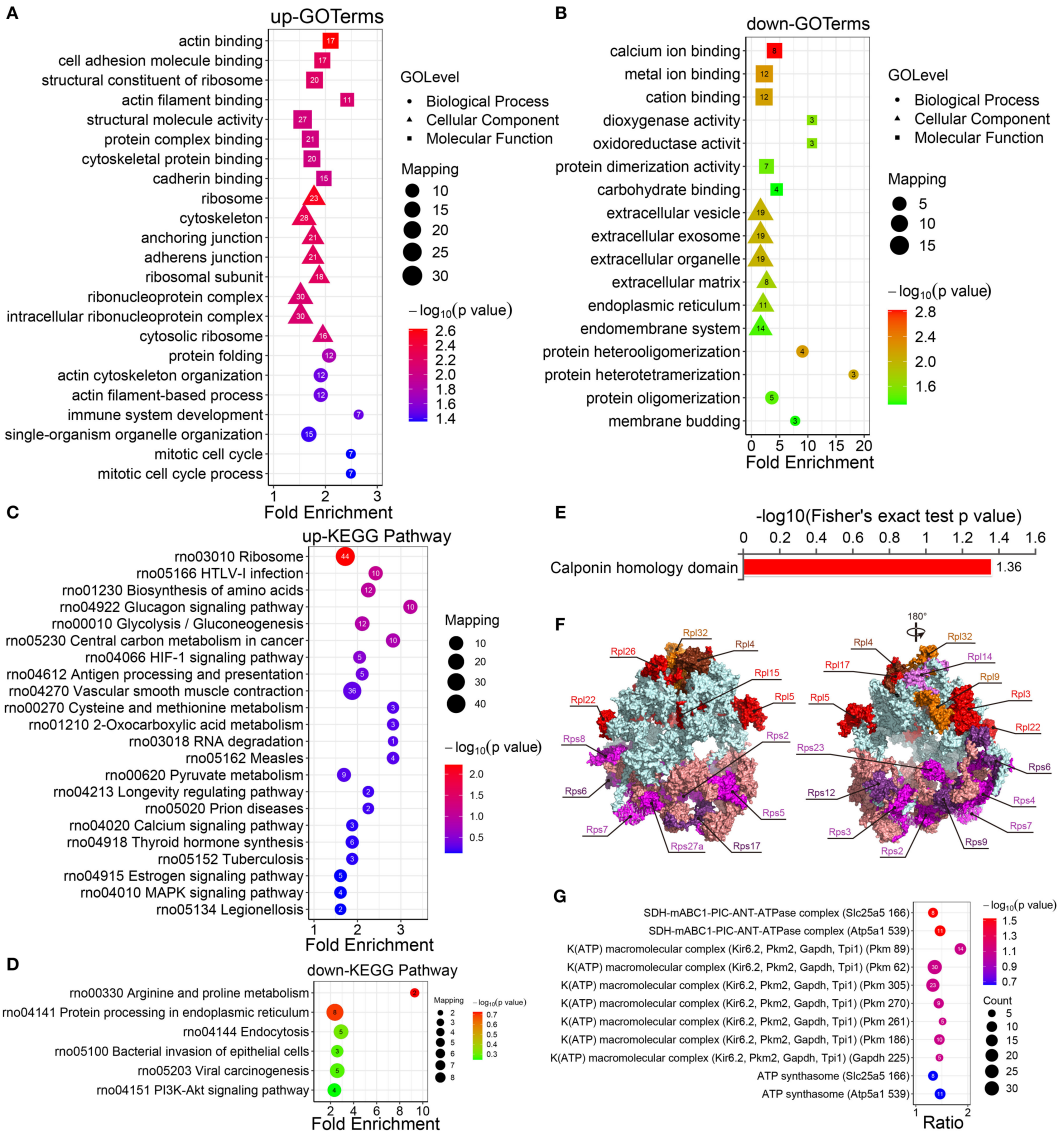
Enrichment analysis of the GO, KEGG pathways, and protein domains of the differentially modified sites. **(A)** The GO enrichment results of the protein corresponding to the upregulated site and **(B)** the protein corresponding to the downregulated site. **(C)** Enrichment analysis of the KEGG pathway of upregulated crotonylated protein. **(D)** Enrichment analysis of the KEGG pathway of downregulated crotonylated protein. **(E)** Enrichment analysis of protein domains of upregulated crotonylated protein. **(F)** The crystal structure of ribosomes and the crotonylated ribosomal proteins (PDB ID: 4V88). **(G)** Predicted functional enrichment of crotonylated protein complexes. The values on the horizontal axis are “Fold Enrichment” significant *p*-values (*p* < 0.05), which are converted to negative logarithms and then plotted as bubble charts. “*p*-value” represents “Fisher's exact *p* value.” Rpl, Ribosomal protein large subunit; Rps, Ribosomal protein small subunit.

To search for the underlying roles of crotonylation in cellular biology, we use the enrichment analysis to categorize crotonylated proteins, which were involved in common pathways or processed as one specific group according to GO databases. GO-based enrichment analysis of the molecular function indicated that the upregulated crotonylated proteins were significantly enriched in the actin-binding, cell adhesion, molecule binding, and structural constituent of ribosomes (Figure 2A). The downregulated crotonylated proteins were noticeably enriched in cation binding, metal ion binding, and calcium ion binding (Figure 2B). A cellular component analysis showed that the upregulated crotonylated proteins were obviously enriched in the cytoskeleton, ribosomes, and anchoring junction (Figure 2A). The downregulated crotonylated proteins were significantly enriched in the extracellular vesicle, extracellular exosome, and extracellular organelle (Figure 2B). The analysis of biological processes indicated that the upregulated crotonylated proteins were enriched in processes related to binding or cytoskeleton, such as actin cytoskeleton organization, protein folding, and actin filament-based process (Figure 2A). The downregulated modified proteins showed a marked difference; however, they were mainly involved in protein heterooligomerization, heterotetramerization, and oligomerization (Figure 2B). GO annotation analyses indicated that crotonylated proteins play an important role in some significant cellular pathways and may function as key regulators in VSMCs.

The KEGG pathway enrichment analysis displayed that the upregulated crotonylated proteins were found to be involved in various pathways, such as ribosomes, HTLV-1 infection, the biosynthesis of amino acids, and glycolysis/gluconeogenesis (Figure 2C). The pathways involved in the downregulated proteins included multiple metabolic pathways, including arginine and proline metabolism, endoplasmic reticulum, endocytosis, bacterial invasion of epithelial cells, and viral carcinogenesis (Figure 2D). We also defined that some metabolic pathways are of significance, which could regulate VSMC phenotypic remodeling, including glycolysis/gluconeogenesis, vascular smooth muscle contraction, and the PI3K-Akt signaling pathway.

The results of a differential protein domain enrichment analysis showed that many upregulated proteins had the same amino acid sequence as the calponin homology (CH) domain (Figure 2E). CH domain is the most common shared amino acid sequence of various actin-binding proteins, which is composed of 6 alpha helices. The CH domain plays a stabilizing role in cytoskeletal dynamics and can activate downstream pathways in signal transduction (35). In this study, there are 6 proteins (transgelin, calponin, microtubule-associated protein RP/EB family member 1, plectin, filamin A, and IQ motif containing GTPase activating protein 1), which contain a CH domain and are extensively crotonylated at multiple sites.

According to the analysis of KEGG pathway enrichment, the ribosome pathway containing 21 ribosomal proteins (9 small subunit ribosomal proteins and 12 large subunit ribosomal proteins) were differential in the presence or absence of PDGF-BB-stimulated VSMCs (Figure 2F; Supplementary Figure S4), which indicated their possible involvement in PDGF-BB-induced VSMC phenotypic remodeling. For the enrichment of protein complexes, we found one statistically significant SDH-mABC1-PIC-ANT-ATPase complex (Figure 2G), which was in the mitochondrial inner membrane and transported K^+^ with similar characteristics of mitoK_ATP_. This implied that differentially expressed proteins may be involved in cell mitochondrial activity and thus affect phenotypic transformation.

To identify the relationship of the functions of proteins with different differential expression fold changes, we divide them into four parts according to their differential expression multiples, including Q1–Q4: Q1 (0 < Ratio ≤ 1/1.5), Q2 (1/1.5 < Ratio ≤ 1/1.3), Q3 (1.3 < Ratio ≤ 1.5), and Q4 (Ratio > 1.5) (Ratio = PDGF/Con) (Supplementary Figure S5A). The statistical results showed that, after PDGF-BB treatment, the number of modification sites in the upregulated proteins significantly increased.

Then, we analyzed every Q group on the functional enrichment and cluster in such aspects, including GO, KEGG, and protein domains. GO enrichment for the Q3 group showed that the upregulated proteins were involved in many biological processes, including the response to hypoxia, the regulation of endocytosis, the regulation of protein metabolic process, positive regulation of cell proliferation, etc. (Supplementary Figure S5B). These upregulated proteins are mainly distributed on the cell surface, cytoskeleton, cytoplasm, and ribosomes (Supplementary Figure S5C). The molecular function analysis of the upregulated proteins showed that they could perform corresponding functions by combining with cell adhesion factors, RNA, cadherin, and ADP (Supplementary Figure S5D). These results suggested that the fold change may be one of the factors that affect the function of modifications.

**Figure 3:**
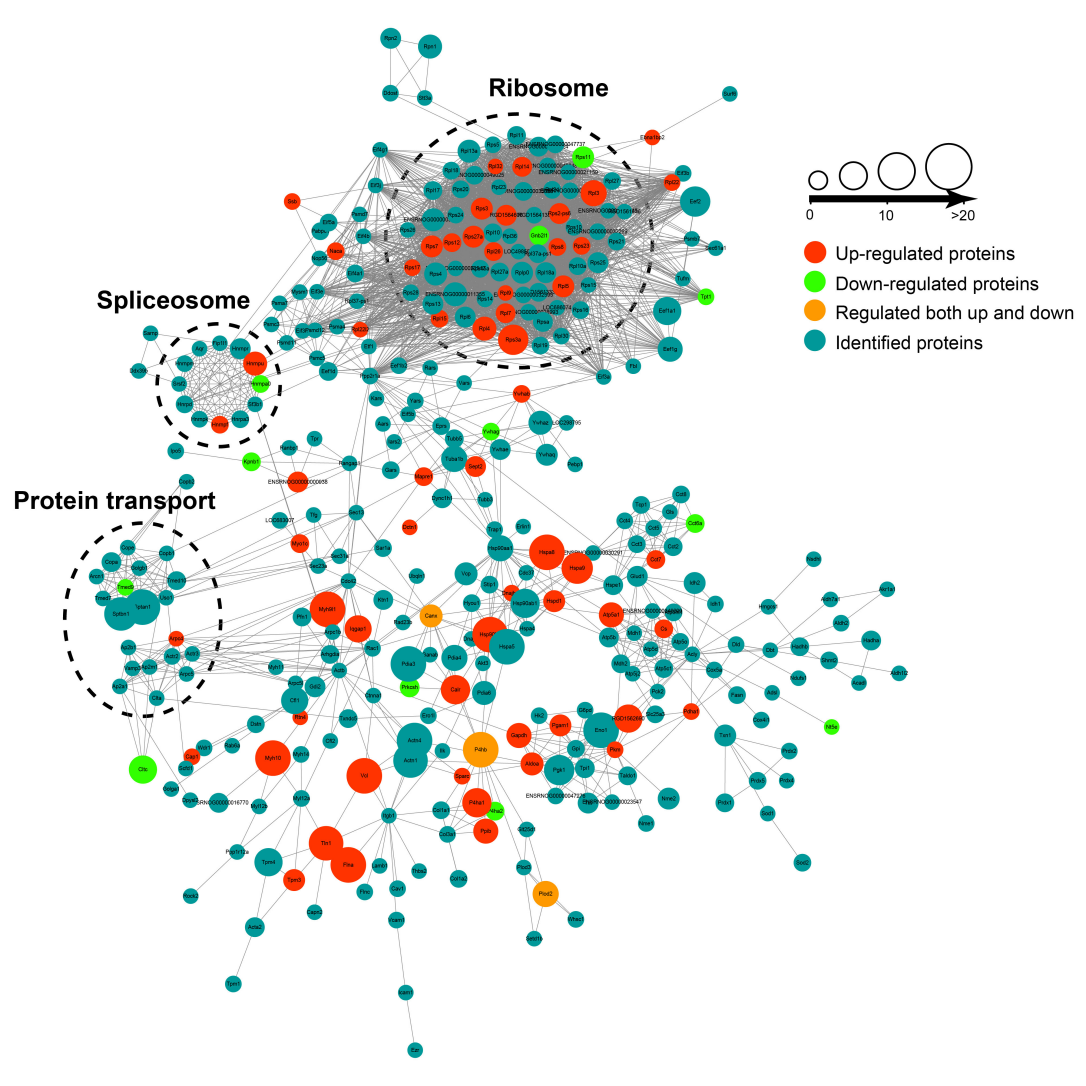
The protein-protein interaction (PPI) network was built according to the STRING database. Interactions belonging to the searched data set were selected.

The protein–protein interaction (PPI) analysis was carried out among the crotonylated proteins based on the STRING database. Functionally clustered crotonylated proteins belonged to multiple protein complexes, including the ribosome, the C complex spliceosome, and protein transport. The interaction network was visualized by Cytoscape (Figure 3).

### Functional Analysis of Lysine Ubiquitination in VSMCs After PDGF-BB Treatment

In this work, the ubiquitylome profile of VSMCs before and after PDGF-BB treatment was investigated and three biological replicates were analyzed for each treatment (Supplementary Figure S6A). Totally, 1,532 ubiquitination sites in 718 proteins were identified, and 1,359 ubiquitination sites in 657 proteins were quantifiable (Table 5). According to a fold change of more than 1.3 or <1/1.3, 124 sites from 84 proteins had been revised upward and 85 sites from 56 proteins were revised downward (Table 6). To confirm the accuracy of the MS data, we performed quality control, and the result showed that our data met the content of subsequent analysis (Supplementary Figure S6B).

**Table 5:** Identification and quantitative statistics of the modification of ubiquitinomics.

	**Identification**	**Quantitative statistics**
Sites	1,532	1,359
Proteins	718	657

**Table 6:** Statistical information of differentially expressed modification level of ubiquitinomics.

**Comparable group**	**Type**	**Up (>1.3)**	**Down (<1/1.3)**
PDGF-vs-Con	Sites	124	85
	Proteins	84	56

**Figure 4:**
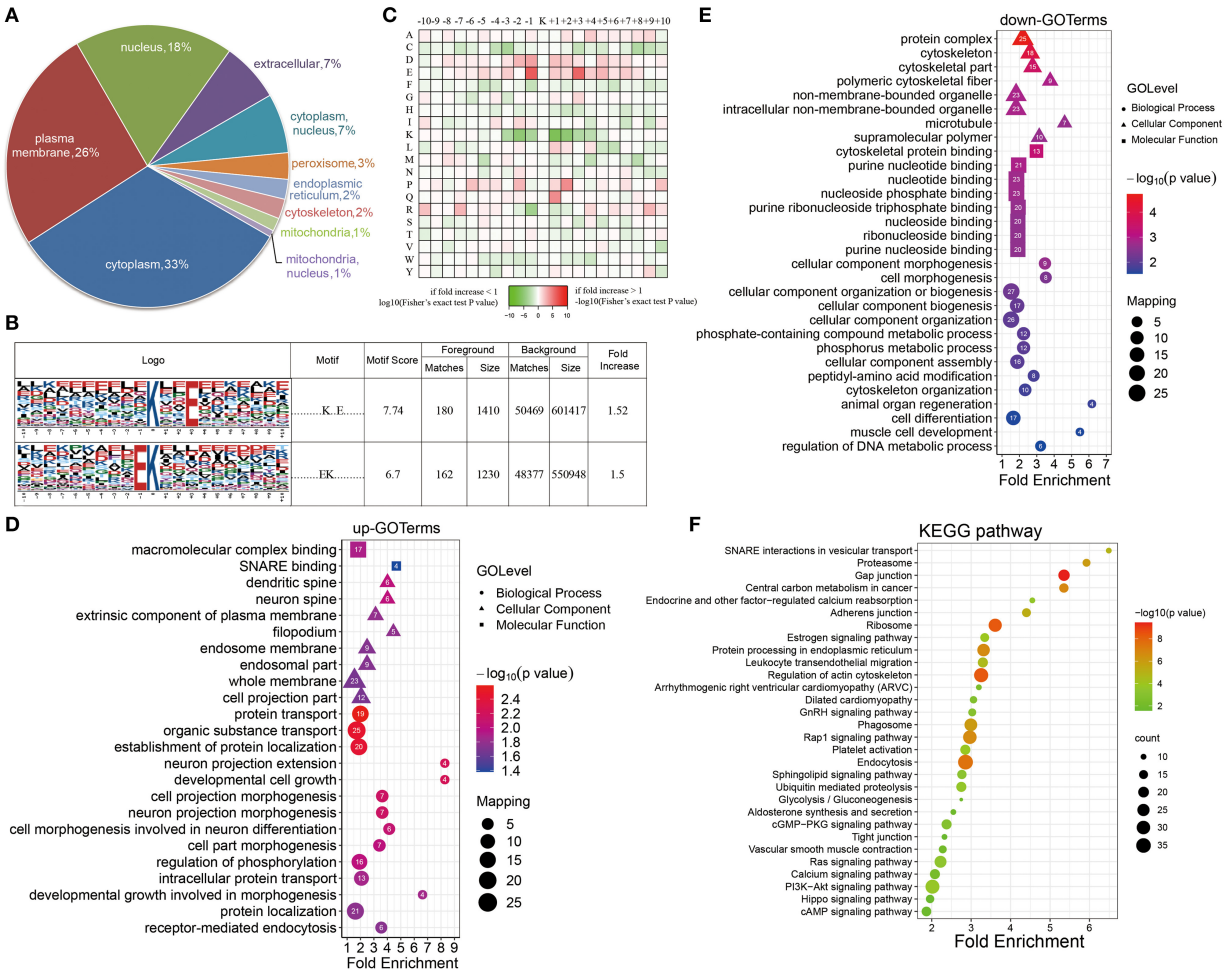
Properties of the quantified ubiquitylome. **(A)** The subcellular localization of the differentially ubiquitination-modified proteins. **(B)** Ubiquitination motifs and the conservation of the ubiquitinated sites. **(C)** A heat map of the amino acid compositions of the lysine ubiquitination sites shows the frequency of different types of amino acids around the ubiquitinated lysine. **(D,E)** GO-based enrichment analysis of proteins with upregulated **(D)** and downregulated **(E)** ubiquitination sites. **(F)** KEGG pathway analysis.

A subcellular localization analysis indicated that differentially expressed ubiquitination-modified proteins were distributed mainly in the plasma membrane, cytoplasm, and nucleus, and a small amount was distributed in the mitochondria, peroxisomes, and cytoskeleton (Figure 4A). To determine the position-specific frequencies of the amino acid residues next to ubiquitination sites, the flanks of these sites were analyzed. We identified two conserved motifs, which were designated as .........EK.........., and ..........K..E....... (Figure 4B). Among them, .........EK.......... has been reported in rice (36) and ..........K..E....... has been reported to be a novel motif in petunia (37). In a previous study, researchers have doubted that the conserved motifs might not exist in mammals because they failed to identify the conserved ubiquitination site motifs in humans (38). Our work for the first time reported the conserved motifs of the ubiquitination sites in rats. Using the statistical analysis of flanking motifs adjacent to the sites, we identified frequent emergence of hydrophilic residue glutamate (E) at the −1 and +3 positions beside the ubiquitination sites (Figure 4C).

To further evaluate ubiquitinated proteins, we classified the differentially regulated ones based on their cellular component, biological processes, and molecular functions (Figures 4D,E). The molecular function analysis showed that the upregulated ubiquitinated proteins were significantly enriched in the macromolecular complex binding. The downregulated ubiquitinated proteins were significantly enriched in the cytoskeleton protein binding and nucleotide binding. For the cellular component analysis, the upregulated ubiquitinated proteins were significantly enriched in membranes, such as an extrinsic component of the plasma membrane, endosome membrane, whole membrane, and filopodium, suggesting that ubiquitination may affect the signaling transmission in the cell membrane. On the contrary, the downregulated ubiquitinated proteins were significantly enriched in the cytoskeleton and microtubule. In the biological process analysis, the upregulated modified proteins took part in varied biological processes, including protein transport, organic substance transport, protein localization, developmental cell growth, and cell morphogenesis. These downregulated modified proteins attached themselves to such processes, including cell morphogenesis, cytoskeleton organization, and cell differentiation.

The KEGG pathway enrichment analysis showed that proteins could function significantly during VSMC phenotypic remodeling, including glycolysis/gluconeogenesis, vascular smooth muscle contraction, RAS signaling pathway, or the PI3K-Akt signaling pathway (Figure 4F). Moreover, the ubiquitination of SM22α, which can promote VSMC survival, was reported in a previous study (13). All these results suggested that protein ubiquitination may possess important roles in the phenotypic remodeling of VSMCs.

### Enrichment Analysis of Crotonylated and Ubiquitinated Proteins

**Figure 5:**
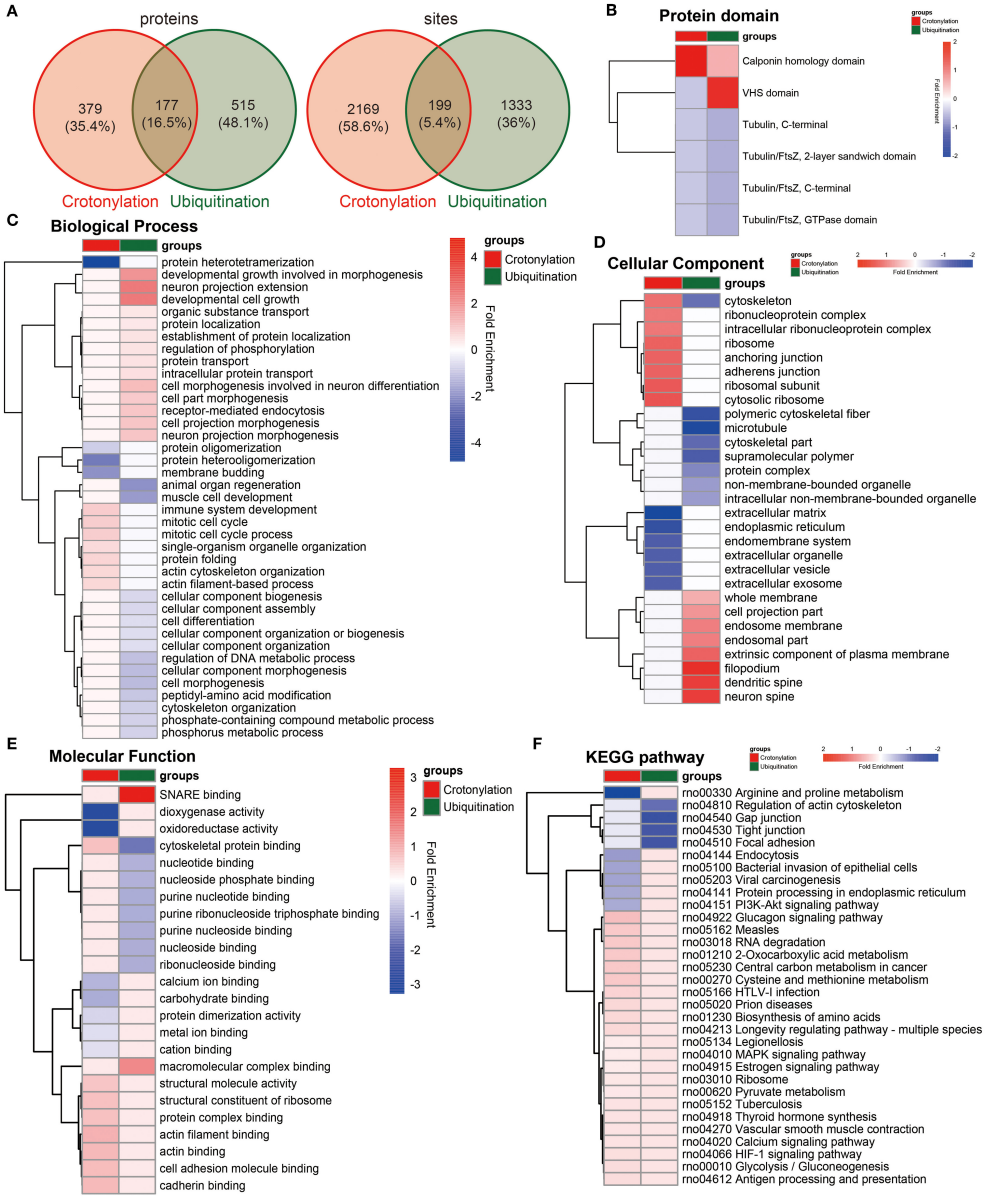
Cross talk results of the quantitative and enrichment analysis between crotonylation and ubiquitination. **(A)** The identified proteins and modification sites of lysine crotonylation and ubiqutination. **(B–F)** The heatmaps show the different and similar enrichment analyses of lysine crotonylation and ubiqutination, including protein domain **(B)**, biological process **(C)**, cellular component **(D)**, molecular function **(E)**, and KEGG pathway **(F)**.

To clarify the relationship between crotonylation and ubiquitination, the cross talk analysis was made on the enrichment analysis and other aspects. Statistical analysis showed that before and after the PDGF-BB stimulation of VSMC, there were 199 sites within the 177 proteins modified by crotonylation and ubiquitination simultaneously (Figure 5A). Then, we compared the enrichment analysis based on the differentially modified proteins in regard to protein domains, GO terms, and KEGG pathways. The protein domain analysis revealed that a significant crotonylated one was the CH domain, which was present in the proteins that could bind actin and affect the cytoskeleton (39) and VHS (Vps-27, Hrs, and STAM) domain in ubiquitination, which was associated with monoubiquitin or polyubiquitin chains' covalent binding with cargo receptors (40) and vesicular tracking after membrane recognition (41) (Figure 5B). In GO terms and KEGG pathways, we found obvious differences and similarities when comparing the two modifications. The crotonylated proteins were extensively involved in actin cytoskeleton organization, and ubiquitinated proteins were mainly involved in cell morphogenesis (Figure 5C). The cellular component of proteins of crotonylation and ubiquitination differed in cytoskeleton obviously (Figure 5D). In the meantime, the molecular function of crotonylated and ubiquitinated proteins was common in structural molecule activity, cadherin binding, and other related binding functions (Figure 5E). The KEGG pathway analysis indicated that crotonylation and ubiquitination co-affect MAPK signaling pathway, pyruvate metabolism, vascular smooth muscle contraction, glycolysis/gluconeogenesis, etc. (Figure 5F). These results suggested that crotonylation and ubiquitination are jointly involved in certain biological functions of cells.

### Cross Talk of PPI Network of Crotonylated and Ubiquitinated Proteins and the Included Enzymes in Glucose Metabolism

**Figure 6:**
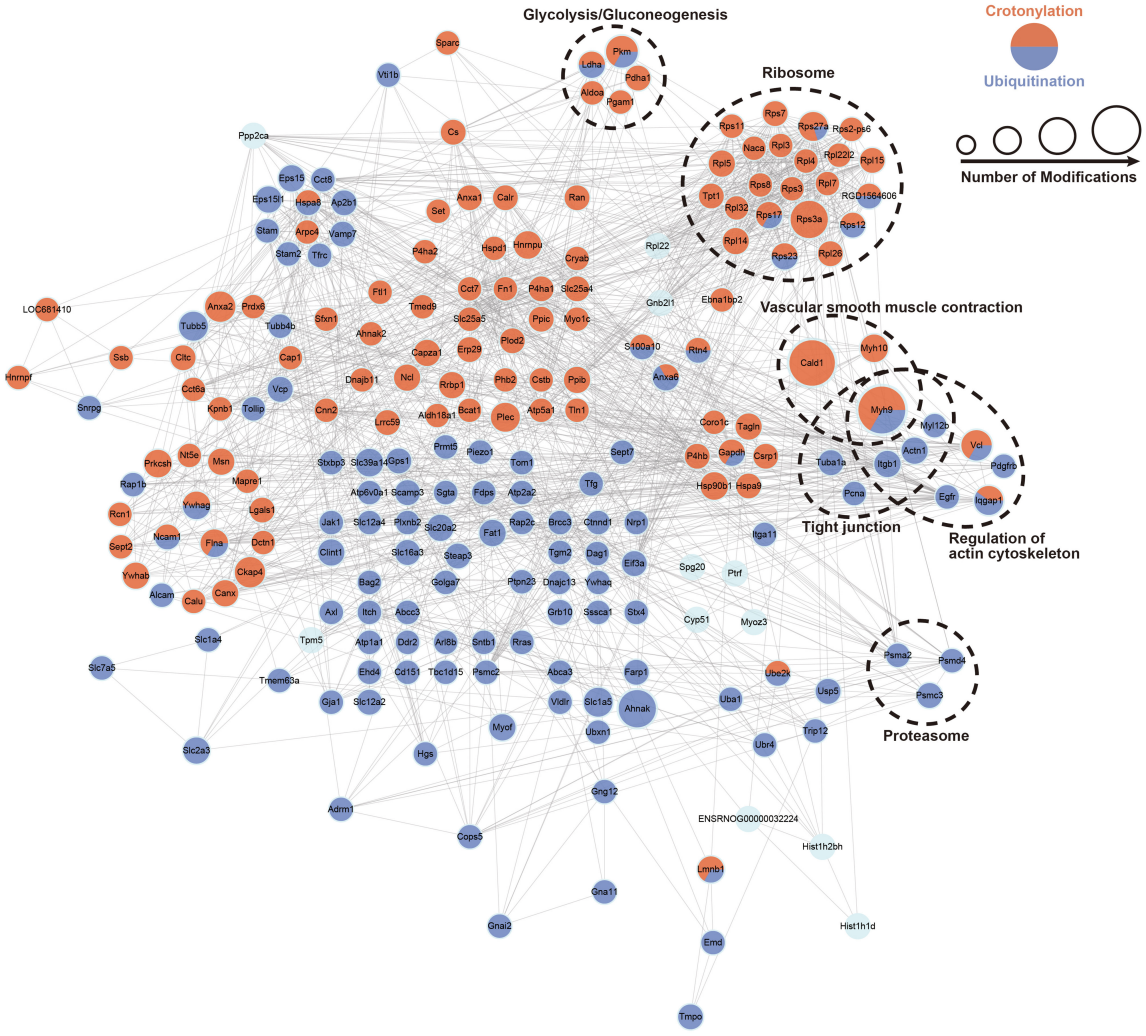
PPI network of a cross talk between crotonylation and ubiquitination was built according to the STRING database. The dotted circles show the functional enrichment of the modified proteins.

We built the PPI network of crotonylated and ubiquitinated proteins, which are significantly differentially expressed based on the STRING database, and clustered the proteins on the strength of MCODE (Figure 6; Supplementary Figure S7). In these clusters, we found a clustering of some important functions, including glycolysis/gluconeogenesis, ribosomes, tight junction, vascular smooth muscle contraction proteasome, and the regulation of actin cytoskeleton. These function clusters were consistent with the KEGG pathway analysis. In these clusters, proteins, which can be both crotonylated and ubiquitinated, would be focused on and could be candidates for future studies, such as Pkm, Ldha, Rps17, Rps27a, Rps12, Rps23, and Myh9. This analysis indicated that crotonylated and ubiquitinated proteins play an important role in cellular bioprocess commonly and possibly have a synergistic effect.

**Figure 7:**
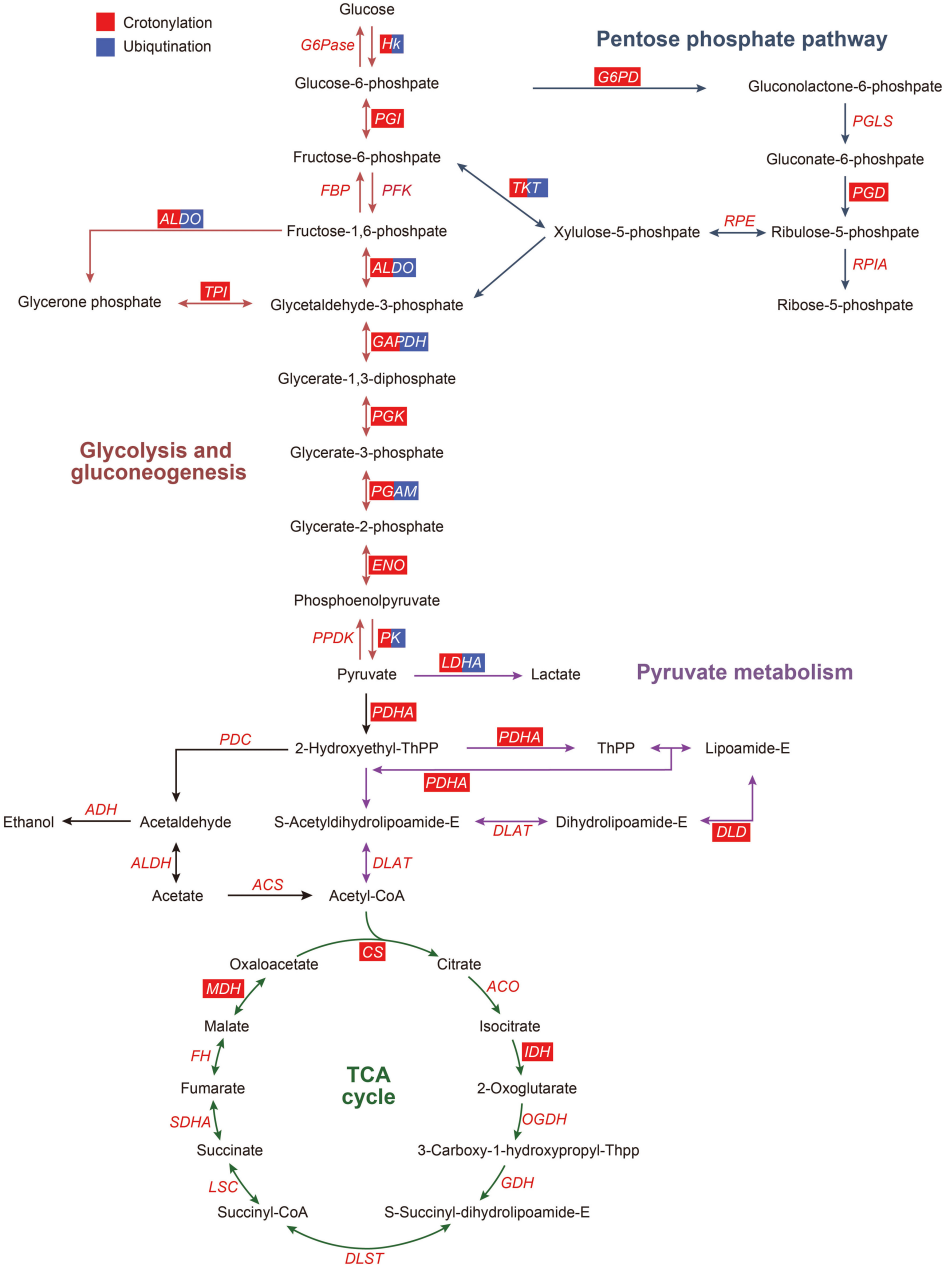
Crotonylated and ubiquitinated enzymes in glycolysis/gluconeogenesis, pentose phosphate pathway, pyruvate metabolism, and TCA cycle. ACO, aconitate hydratase; ACS, acetyl coenzyme A synthetase; ADH, alcohol dehydrogenase; ALDH, aldehyde dehydrogenase; ALDO, aldolase; CS, citrate synthase; DLAT, dihydrolipoamide acetyltransferase (pyruvate dehydrogenase E2 component); DLD, dihydrolipoamide dehydrogenase; DLST, dihydrolipoamide succinyltransferase (2-oxoglutarate dehydrogenase E2 component); ENO, enolase; FBP, fructose-biphosphatase; HK, hexokinase; IDH, isocitrate dehydrogenase; G6Pase, glucose-6-phosphatase; G6PD, glucose-6-phosphate dehydrogenase; GAPDH, glyceraldehyde phosphate dehydrogenase; LDHA, lactate dehydrogenase A; LSC, succinyl-CoA synthetase; MDH, malate dehydrogenase; OGDH, 2-oxoglutarate dehydrogenase E1 component; PDC, pyruvate dehydrogenase complex; PDH, pyruvate dehydrogenase; PFKA, phosphofructokinase; PK, pyruvate kinase; PGAM, phosphoglycerate mutase; PGD, 6-phosphogluconate dehydrogenase, decarboxylating 2; PGI, phosphohexose isomerase; PGK, phosphoglycerate kinase; PGLS, 6-phosphogluconolactonase; RPIA, ribose-5-phosphate isomerase; RPE, Ribulosephosphate 3-epimerase; TCA cycle, tricarboxylic acid cycle; SDHA, succinate dehydrogenase; TKT, transketolase; TPI, triose phosphate isomerase. The red box represents the enzymes modified by crotonylation, and the blue box represent the enzymes modified by ubiquitination.

According to the KEGG pathway analysis and function clustering based on MCODE, glycolysis/gluconeogenesis was highlighted (Figure 7). Most of the enzymes involved in glycolysis were the significant ones as they were crotonylated and ubiquitinated. Among them, one of the key enzymes, hexokinase, had two crotonylation-modified sites and one ubiquitination-modified site. Another key enzyme, PK, had 12 crotonylation-modified sites and 4 ubiquitination-modified sites. Other important enzymes involved in glycolysis also had varying degrees of modifications. These results suggested that a possible change in glycolysis is obvious when it was affected by crotonylation and ubiquitination.

## Discussion

Histone crotonylation, a type of lysine modification, is newly found in mammalian cells, and this modification is significantly enriched in gene promoters and enhancers (19). Previous studies have found that histone crotonylation can inhibit the expression of pro-growth genes (42) and sex chromosome-linked genes (43). Recently, although there are more and more articles on the crotonylation of nonhistone proteins, the scope of these studies is very narrow, and there is no such report in the cardiovascular field. However, the crotonylation of lysine in nonhistone proteins in VSMCs is not reported so far.

To identify whether the crotonylation of lysine occurs in VSMCs and also to explore its possible function within cellular bioprocesses, a comprehensive crotonylomics was realized by combing high-resolution LC-MS/MS with another detective technology of immune-affinity purification with high sensitivity. The MS results showed that 2,138 lysine crotonylation sites were identified in 534 proteins, which were the most abundant ones in the nonhistone protein acylation proteome of VSMCs so far. These crotonylated proteins were involved in multiple biological processes, including glucose metabolism, amino acid biosynthesis, protein folding, and RNA degradation according to the GO and KEGG pathway analysis. In coordination with the PPI network analysis, the enrichment analysis also showed a widespread interaction between crotonylated proteins and the clustering of their functions, such as ribosomes and spliceosomes.

Lysine acetylated proteins are involved in many glucose-metabolic pathways, such as carbon metabolism, glycolysis/gluconeogenesis, the tricarboxylic acid (TCA) cycle, pentose phosphate pathway, conservative in eukaryotes, and also in prokaryotes (14, 15, 44–46). In this paper, we found that numerous enzymes involved in the glucose metabolic pathways could be modified on lysine by crotonyl. In PDGF-BB-stimulated VSMCs, the enzymes that undergo crotonylation were mainly those enzymes involved in glycolysis. There were 50% (8/16) enzymes in the glycolysis/gluconeogenesis in which crotonylation occurs at a great many sites, with an average of 10. For example, PK, one of the key glycolysis metabolic enzymes, was crotonylated at 12 different sites. The key lysine residue of PKM, K305, was acetylated and could result in decreased PKM activity (47), which also showed the upregulated crotonylation modification. This indicated that crotonylation possibly modulates the activity of PKM in cooperation and coordination with acetylation. Moreover, 30% (3/10) of the enzymes involved in pyruvate metabolism were modified by crotonylation and these enzymes have not been reported on acetylation. These results could give us a conclusion that lysine crotonylation of lysines might play an important irreplaceable role in the regulation of glycolysis/gluconeogenesis metabolism.

Vascular smooth muscle cells show significant phenotypic and functional changes after vascular injury. In the presence of vascular injury, the contractile phenotype of VSMCs can be converted into the proliferative phenotype, and contraction markers showed a decrease accompanied by this change. Other proinflammatory mediators, which could induce proliferation and chemotaxis, would gradually increase. Activated VSMCs could proliferate and migrate significantly, thereby accelerating post-injury repair of blood vessels. In this study, lysine crotonylation of 5 VSMC contractile markers extensively occurred at multiple sites. For example, caldesman1 was crotonylated at 16 sites and myosin9 at 15 sites. Moreover, transgelin was also crotonylated at 3 lysine sites. These results suggest that lysine crotonylation is likely to be involved in regulating VSMC contraction.

Proteins are biological macromolecules that perform a wide range of functions in living organisms. Amino acids are the basic units of protein. The synthesis and degradation of amino acids were related to the lysine crotonylation shown in our research, such as arginine, proline, cysteine, and methionine. Protein synthesis was also affected by lysine crotonylation. In our study, we found 21 ribosome subunits that were crotonylated. The crotonylated ribosome proteins participate in the stabilization of the structure and the formation of peptides. Bhushan et al. demonstrated that nascent polypeptide chains are connected in a set of discrete tunnel constituents, including the extensions of L4 and L17 (L22), and rRNA nucleotides U2585, A2062, A2058, and A751 (48). The L4 globular domain promoted the folding of early rRNA, and the internal loop could aggregate and stabilize the domain of the 60S subunit functional active site (49). Also, S3 could bind to a receptor for activated c-kinase (RACK1, Asc1 in yeast) through the C-terminal extension sequence to stabilize protein synthesis and cell growth (50). HSP60 and HSP90 families are chaperones, which give assistance to the amino acid folding progress. They were extensively crotonylated after the activation of VSMCs, and this result indicated that lysine crotonylation might affect the folding process. In this study, we found ubiquitin-40S ribosomal protein S27a (Rps27a) was modified through crotonylation. These results thus imply that the crotonylation of lysine could regulate the bioprocesses, including protein synthesis, folding process, and ubiquitination degradation.

Together with crotonylome, nonhistone protein ubiquitylnome of VSMCs was performed by LC-MS/MS accompanied by immune-affinity purification with high sensitivity. We finally obtained the result that showed 1,359 quantitative sites in 657 proteins altogether. These ubiquitinated proteins were mainly involved in cell morphogenesis, cytoskeleton, and cellular component organization, proteasome, ribosome, and the regulation of actin cytoskeleton based on the analysis of GO and KEGG pathways.

In the past research, there are articles summarizing the cross talk of multiple PTMs in *Candida albicans* (51), developing rice seeds (52), yeast sporulation, and mouse spermatogenesis (53). In VSMC, little is known about the cross talk of multi-PTMs. In our work, we found some proteins and a functional overlap in crotonylation and ubiquitination. The results of the cross talk analysis showed that 199 sites of 177 proteins could be modified by crotonyl and ubiquitin simultaneously. The obviously changeable GO and KEGG pathway included cytoskeleton, cytoskeleton protein binding, actin-binding, and the regulation of actin cytoskeleton. These cellular component and function analyses indicated that crotonylation and ubiquitination were involved in cytoskeleton remodeling and the modulation of the phenotypic transformation. In the PPI network analysis with clustering of functions, we found that myosin heavy chain 9 (MYH9) containing the upregulated 18 crotonylated lysine sites and the downregulated 3 ubiquitinated sites was the point of intersection of tight junction, VSMC contraction, and the regulation of actin cytoskeleton. MYH9 is a widespread cytoplasmic myosin and is involved in the translocation of the skeleton proteins (54). Moreover, some studies showed that MYH9 was upregulated in gastric cancer, which could promote the transcription of β-catenin (55) and could be upregulated in esophageal squamous cell carcinoma (ESCC) facilitating cell metastasis (56). Also, MYH9 could bind with and degrade GSK3β through ubiquitin, therefore β-catenin was downregulated to induce the epithelial-mesenchymal transition in hepatocellular carcinoma (57). However, the function of MYH9 in phenotypic remodeling remains unknown, which provides a possible direction for subsequent research.

In our previous study, the activity of G6PD could be affected by SM22α ubiquitination (13), which indicated a possible relation between PTM and phenotypic remodeling. Combining with the enrichment analysis of the GO and KEGG pathway and PPI network, we noticed that enzymes of the glucose metabolism pathways, including glycolysis/gluconeogenesis, pyruvate metabolism, pentose phosphate pathway, and TCA cycle, were modified extensively by crotonyl and ubiquitin. The modification of the enzymes might modulate the pathways directly or indirectly. Among these pathways, the enzymes of glycolysis were mostly both crotonylated and ubiquitinated. Meanwhile, in other pathways, crotonylation is more widely distributed, suggesting its significance in the modulation of glucose metabolism in VSMCs. Of course, our analysis is partial and limited. In this study, we just only combine our own data to dig into more possible interesting phenomena, and omics of other types of PTMs on nonhistone proteins are rarely studied in VSMC.

In summary, our study gave an overview of the profiling analysis of global nonhistone protein lysine crotonylome and ubiquitylome in VSMCs for the first time. The results showed more prevalence of crotonylation than ubiquitination and their cross talk analysis focusing on GO, KEGG, and PPI suggested that their biological significance was associated with glycolysis, VSMC contraction, or cellular skeleton modulation. The cross talk of crotonylation and ubiquitination in glycolysis is possibly a novel mechanism underlying VSMC phenotypic remodeling. Related crotonylated and ubiquitinated proteins will be the emphasis for further functional studies.

## Data Availability Statement

The datasets presented in this study can be found in online repositories. The names of the repository/repositories and accession number(s) can be found below: PRIDE database; PXD029070.

## Ethics Statement

This animal study was reviewed and approved by the Institutional Animal Care and Use Committee of Hebei Medical University.

## Author Contributions

S-HC contributed to organizing and analyzing the data, making the figures, and writing the most of the manuscript. Z-HC wrote the part of ubiquitination. LY, H-MJ, and R-YM contributed to organizing the data and figures. L-HD contributed to analyzing and interpreting the data, writing and revising the manuscript, and obtaining the funding. All authors contributed to the article and approved the submitted version.

## Funding

This study was supported by grants from the National Natural Science Foundation of China (81670394), the Hebei Natural Science Foundation (C2019206022), and the Hebei Scientific and Technological Research Projects (ZD2020302).

## Conflict of Interest

The authors declare that the research was conducted in the absence of any commercial or financial relationships that could be construed as a potential conflict of interest.

## Publisher's Note

All claims expressed in this article are solely those of the authors and do not necessarily represent those of their affiliated organizations, or those of the publisher, the editors and the reviewers. Any product that may be evaluated in this article, or claim that may be made by its manufacturer, is not guaranteed or endorsed by the publisher.
