# Supplementary material for: Dynamics and Functional Interplay of Nonhistone Lysine Crotonylome and Ubiquitylome in Vascular Smooth Muscle Cell Phenotypic Remodeling
Source: Front Cardiovasc Med. 2022 Mar 8;9:783739. doi: 10.3389/fcvm.2022.783739 (PMC8964401; doi:10.3389/fcvm.2022.783739)
Supplement: Supplementary file 1 [file Data_Sheet_1.docx]

**Supplementary Information**

**Experimental Procedures**

**1. Preparation of VSMC extraction for LC-MS**

VSMCs with or without PDGF-BB stimulated were sonicated three times on ice using a high intensity ultrasonic processor (Scientz) in lysis buffer (8 M urea, 3 μM TSA, 50 mM NAM and 1% Protease Inhibitor Cocktail). The remaining debris was removed by centrifugation at 12,000 g at 4 °C for 10 min. Finally, the supernatant was collected and the protein concentration was determined with BCA kit according to the manufacturer’s instructions.

**2. Trypsin Digestion**

For digestion, the protein solution was reduced with 5 mM dithiothreitol for 30 min at 56 °C and alkylated with 11 mM iodoacetamide for 15 min at room temperature in darkness. The protein sample was then diluted by adding 100 mM NH_4_HCO_3_ to urea concentration less than 2M. Finally, trypsin was added at 1:50 trypsin-to-protein mass ratio for the first digestion overnight and 1:100 trypsin-to-protein mass ratio for a second 4 h-digestion.

**3. HPLC Fractionation**

The tryptic peptides were fractionated into fractions by high pH reverse-phase HPLC using Thermo Betasil C18 column (5 μm particles, 10 mm ID, 250 mm length). Briefly, peptides were first separated with a gradient of 8% to 32% acetonitrile (pH 9.0) over 60 min into 60 fractions. Then, the peptides were combined into 10 fractions and dried by vacuum centrifuging.

**4. Affinity Enrichment of crotonylated peptides**

To enrich crotonylated peptides, tryptic peptides dissolved in NETN buffer (100 mM NaCl, 1 mM EDTA, 50 mM Tris-HCl, 0.5% NP-40, pH 8.0) were incubated with pre-washed antibody beads (PTM Bio) at 4°C overnight with gentle shaking. Then the beads were washed four times with NETN buffer and twice with H_2_O. The bound peptides were eluted from the beads with 0.1% trifluoroacetic acid. Finally, the eluted fractions were combined and vacuum-dried. For LC-MS/MS analysis, the resulting peptides were desalted with C18 ZipTips (Millipore) according to the manufacturer’s instructions.

**5. LC-MS/MS Analysis**

The tryptic peptides were dissolved in 0.1% formic acid (solvent A), directly loaded onto a home-made reversed-phase analytical column (15-cm length, 75 μm i.d.). The gradient was comprised of an increase from 6% to 23% solvent B (0.1% formic acid in 98% acetonitrile) over 26 min, 23% to 35% in 8 min and climbing to 80% in 3 min then holding at 80% for the last 3 min, all at a constant flow rate of 400 nL/min on an EASY-nLC 1000 UPLC system. The peptides were subjected to NSI source followed by tandem mass spectrometry (MS/MS) in Q ExactiveTM Plus (Thermo) coupled online to the UPLC. The electrospray voltage applied was 2.0 kV. The m/z scan range was 350 to 1800 for full scan, and intact peptides were detected in the Orbitrap at a resolution of 70,000. Peptides were then selected for MS/MS using NCE setting as 28, and the fragments were detected in the Orbitrap at a resolution of 17,500. A data-dependent procedure that alternated between one MS scan followed by 20 MS/MS scans with 15.0s dynamic exclusion. Automatic gain control (AGC) was set at 5E4.

**6. Database Search**

The resulting MS/MS data were processed using Maxquant search engine (v.1.5.2.8). Tandem mass spectra were searched against **UniProt** database concatenated with reverse decoy database. Trypsin/P was specified as cleavage enzyme, allowing up to 4 missing cleavages. The mass tolerance for precursor ions was set as 20 ppm in First search and 5 ppm in Main search, and the mass tolerance for fragment ions was set as 0.02 Da. Carbamidomethyl on Cys was specified as fixed modification and contractile and oxidation on Met were specified as variable modifications. Label-free quantification method was LFQ, FDR was adjusted to < 1% and the minimum score for modified peptides was set > 40.

**7. Cell culture and treatment**

VSMC was isolated from the thoracic aorta of 80-100 g male Sprague-Dawley rats as previously described. VSMCs were grown in low glucose Dulbecco’s-modified Eagle’s medium (DMEM) (Invitrogen, US) with 10% fetal bovine serum (FBS), 100 U/ml pencillin, and 100 ug/ml streptomycin. The VSMCs were maintained at 37°C in a humidified atmosphere containing 5% CO_2_, and only passages 3 to 5 cells at 70-80% confluence were used in the experiments, except if stated otherwise. HEK293 cells were cultured in high glucose DMEM containing 10% FBS. This study was performed via a protocol approved by the Institutional Animal Care and Use Committee of Hebei Medical University, in accordance with the Guide for the Care and Use of Laboratory Animals, and the Hebei Medical University Clinical Research Ethics Committee.

**8. Plasmids and antibodies**

The cDNA of PKM and LDHA were amplified by PCR and subcloned into pcDNA-HA vector (biyuntian). The following antibodies were used in the expriments: Pan-Kcr (PTM Biol 501), FLAG and HA.

**9. Co-immunoprecipitation**

Cells were lysed in NP40 buffer (50 mM Tris-HCl, pH 7.4, 150 mM NaCl, 1% NP-40, 1 mM EDTA, 10 mM sodium butyrate) containing protease inhibitors for 30 min on ice. Then, lysates were incubated with relevant antibody overnight at 4 °C. And 50 μl protein A/G agarose beads were added and incubated for 2 h at 4 °C. After washed with NP40 buffer for three times, the immunoprecipitated complexes were subjected to SDS-PAGE and immunoblotted with the indicated antibodies.

**10. Immunofluorescence**

Cells were cultivated on coverslips prior to experiment. Cells were pre-washing with PBS for twice and fixed with 4% paraformaldehyde for 15 min at room temperature. Then the cells were permeabilized with 0.1% NP-40 for 15 min. Permeabilized cells were blocked with 5% BSA in PBS for 1 h. After washed with PBS three times, cells were incubated overnigt at 4 °C with pan-Kcr antibody. After washed with PBS for three times, cells were incubated with FITC-goat anti-rabbit IgG for 1 h at 4 °C. Finally, cells were stained with DAPI and mounted to glass slides. Microscopic imaged were captured by confocal laser-scanning microscope (Leica).

Bioinformatics Methods

1. Annotation Methods

GO Annotation

The Gene Ontology, or GO, is a major bioinformatics initiative to unify the representation of gene and gene product attributes across all species. More specifically, the project aims to:

1. Maintain and develop its controlled vocabulary of gene and gene product attributes;

2. Annotate genes and gene products, and assimilate and disseminate annotation data;

3. Provide tools for easy access to all aspects of the data provided by the project.

The ontology covers three domains:

1. Cellular component: A cellular component is just that, a component of a cell, but with the proviso that it is part of some larger object; this may be an anatomical structure (e.g. rough endoplasmic reticulum or nucleus) or a gene product group (e.g. ribosome, proteasome or a protein dimer).

2. Molecular function: Molecular function describes activities, such as catalytic or binding activities, that occur at the molecular level. GO molecular function terms represent activities rather than the entities (molecules or complexes) that perform the actions, and do not specify where or when, or in what context, the action takes place.

3. Biological process: A biological process is series of events accomplished by one or more ordered assemblies of molecular functions. It can be difficult to distinguish between a biological process and a molecular function, but the general rule is that a process must have more than one distinct steps.

Gene Ontology (GO) annotation proteome was derived from the UniProt-GOA database (www.http://www.ebi.ac.uk/GOA/). Firstly, Converting identified protein ID to UniProt ID and then mapping to GO IDs by protein ID. If some identified proteins were not annotated by UniProtGOA database, the InterProScan soft would be used to annotated protein’s GO functional based on protein sequence alignment method. Then proteins were classified by Gene Ontology annotation based on three categories: biological process, cellular component and molecular function.

Domain Annotation

A protein domain is a conserved part of a given protein sequence and structure that can evolve, function and exist independently of the rest of the protein chain. Each domain forms a compact three-dimensional structure and often can be independently stable and folded. Many proteins consist of several structural domains. One domain may appear in a variety of differentially modified proteins. Molecular evolution uses domains as building blocks and these may be recombined in different arrangements to create proteins with different functions. Domains vary in length from between about 25 amino acids up to 500 amino acids in length. The shortest domains such as zinc fingers are stabilized by metal ions or disulfide bridges. Domains often form functional units, such as the calcium-binding EF hand domain of calmodulin. Because they are independently stable, domains can be "swapped" by genetic engineering between one protein and another to make chimeric proteins. Identified proteins domain functional description were annotated by InterProScan (a sequence analysis application) based on protein sequence alignment method, and the InterPro domain database was used. InterPro (http://www.ebi.ac.uk/interpro/) is a database that integrates diverse information about protein families, domains and functional sites, and makes it freely available to the public via Web-based interfaces and services. Central to the database are diagnostic models, known as signatures, against which protein sequences can be searched to determine their potential function. InterPro has utility in the large-scale analysis of whole genomes and meta-genomes, as well as in characterizing individual protein sequences.

KEGG Pathway Annotation

KEGG connects known information on molecular interaction networks, such as pathways and complexes (the "Pathway" database), information about genes and proteins generated by genome projects (including the gene database) and information about biochemical compounds and reactions (including compound and reaction databases). These databases are different networks, known as the "protein network", and the "chemical universe" respectively. There are efforts in progress to add to the knowledge of KEGG, including information regarding ortholog clusters in the KEGG Orthology database. KEGG Pathways mainly including: Metabolism, Genetic Information Processing, Environmental Information Processing, Cellular Processes, Rat Diseases, Drug development. Kyoto Encyclopedia of Genes and Genomes (KEGG) database was used to annotate protein pathway. Firstly, using KEGG online service tools KAAS to annotated protein’s KEGG database description. Then mapping the annotation result on the KEGG pathway database using KEGG online service tools KEGG mapper.

1. Subcellular Localization

The cells of eukaryotic organisms are elaborately subdivided into functionally distinct membrane bound compartments. Some major constituents of eukaryotic cells are: extracellular space, cytoplasm, nucleus, mitochondria, Golgi apparatus, endoplasmic reticulum (ER), peroxisome, vacuoles, cytoskeleton, nucleoplasm, nucleolus, nuclear matrix and ribosomes. Bacteria also have subcellular localizations that can be separated when the cell is fractionated. The most common localizations referred to include the cytoplasm, the cytoplasmic membrane (also referred to as the inner membrane in Gram-negative bacteria), the cell wall (which is usually thicker in Gram-positive bacteria) and the extracellular environment. Most Gramnegative bacteria also contain an outer membrane and periplasmic space. Unlike eukaryotes, most bacteria contain no membrane-bound organelles, however there are some exceptions. There, we used wolfpsort a subcellular localization predication soft to predict subcellular localization. Wolfpsort is an updated version of PSORT/PSORT II for the prediction of eukaryotic sequences. Special for protokaryon species, Subcellular localization prediction soft CELLO was used.

2. Motif Analysis

Soft motif-x was used to analysis the model of sequences constituted with amino acids in specific positions of modify-21-mers (10 amino acids upstream and downstream of the site， but phosphorylation with modify-13-mers that 6 amino acids upstream and downstream of the site) in all protein sequences. And all the database protein sequences were used as background database parameter, other parameters with default.

3. Functional Enrichment

Enrichment of Gene Ontology analysis

Proteins were classified by GO annotation into three categories: biological process, cellular compartment and molecular function. For each category, a two-tailed Fisher’s exact test was employed to test the enrichment of the differentially modified protein against all identified proteins. The GO with a corrected p-value < 0.05 is considered significant.

Enrichment of pathway analysis

Encyclopedia of Genes and Genomes (KEGG) database was used to identify enriched pathways by a two-tailed Fisher’s exact test to test the enrichment of the differentially modified protein against all identified proteins. The pathway with a corrected p-value < 0.05 was considered significant. These pathways were classified into hierarchical categories according to the KEGG website.

Enrichment of protein domain analysis

For each category proteins, InterPro (a resource that provides functional analysis of protein sequences by classifying them into families and predicting the presence of domains and important sites) database was researched and a two-tailed Fisher’s exact test was employed to test the enrichment of the differentially modified protein against all identified proteins. While p-value < 0.05 was considered significant.

4. Enrichment-based Clustering

For further hierarchical clustering based on differentially modified protein functional classification (such as: GO, Domain, Pathway, Complex). We first collated all the categories obtained after enrichment along with their P values, and then filtered for those categories which were at least enriched in one of the clusters with P value <0.05. This filtered P value matrix was transformed by the function x = -log10 (P value). Finally， these x values were transformed for each functional category. These z scores were then clustered by one-way hierarchical clustering (Euclidean distance, average linkage clustering) in Genesis. Cluster membership was visualized by a heat map using the “heatmap.2” function from the “gplots” R-package.

5. Protein-protein Interaction Network

All identified protein name identifiers were searched against the STRING database version 10.5 for protein-protein interactions. Only interactions between the proteins belonging to the searched data set were selected, thereby excluding external candidates. STRING defines a metric called “confidence score” to define interaction confidence; we fetched all interactions that had a confidence score ≥ 0.9 (high confidence). Interaction network form STRING was visualized in Cytoscape. The oretical clustering algorithm, molecular complex detection (MCODE) was utilized to analyze densely connected regions. MCODE is part of the plug-in tool kit of the network analysis and visualization software Cytoscape.

**Supplementary Figures**


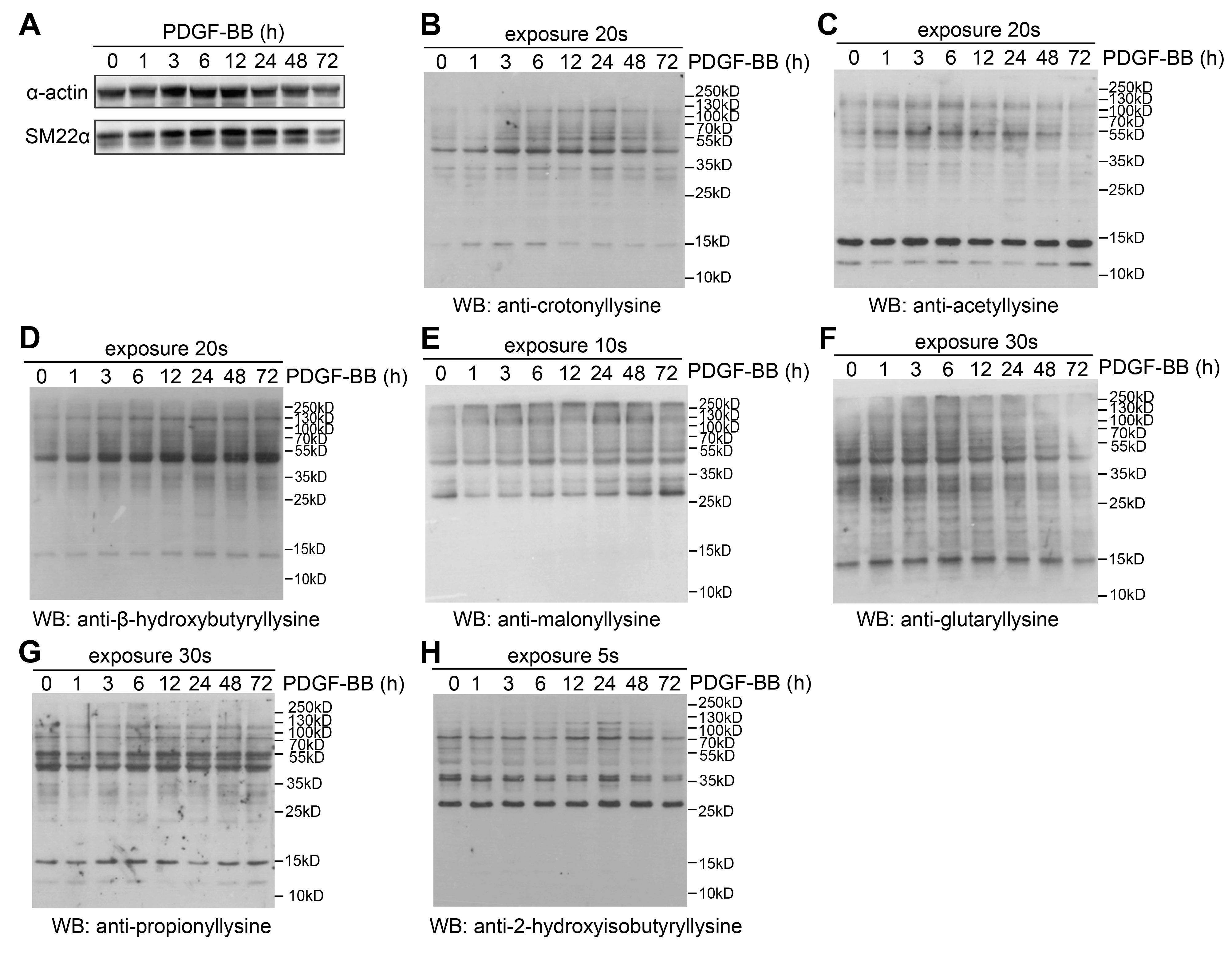


**Supplementary Figure 1.** Detection of multiple acylation levels of whole protein at multiple time points. **(A)** The expression level of the smooth muscle contractile proteins SM α-actin and SM22α. **(B-H)** The pan- crotonylated /acetylated/β-hydroxybutyrylated/malonylated/ glutarylated/propionylated/2-hydroxyisobutyrylated level in VSMCs upon PDGF-BB treatment for different time. VSMCs were incubated with PDGF-BB (10 ng/ml) for 0,1, 3, 6, 12, 24, 48 and 72 h.


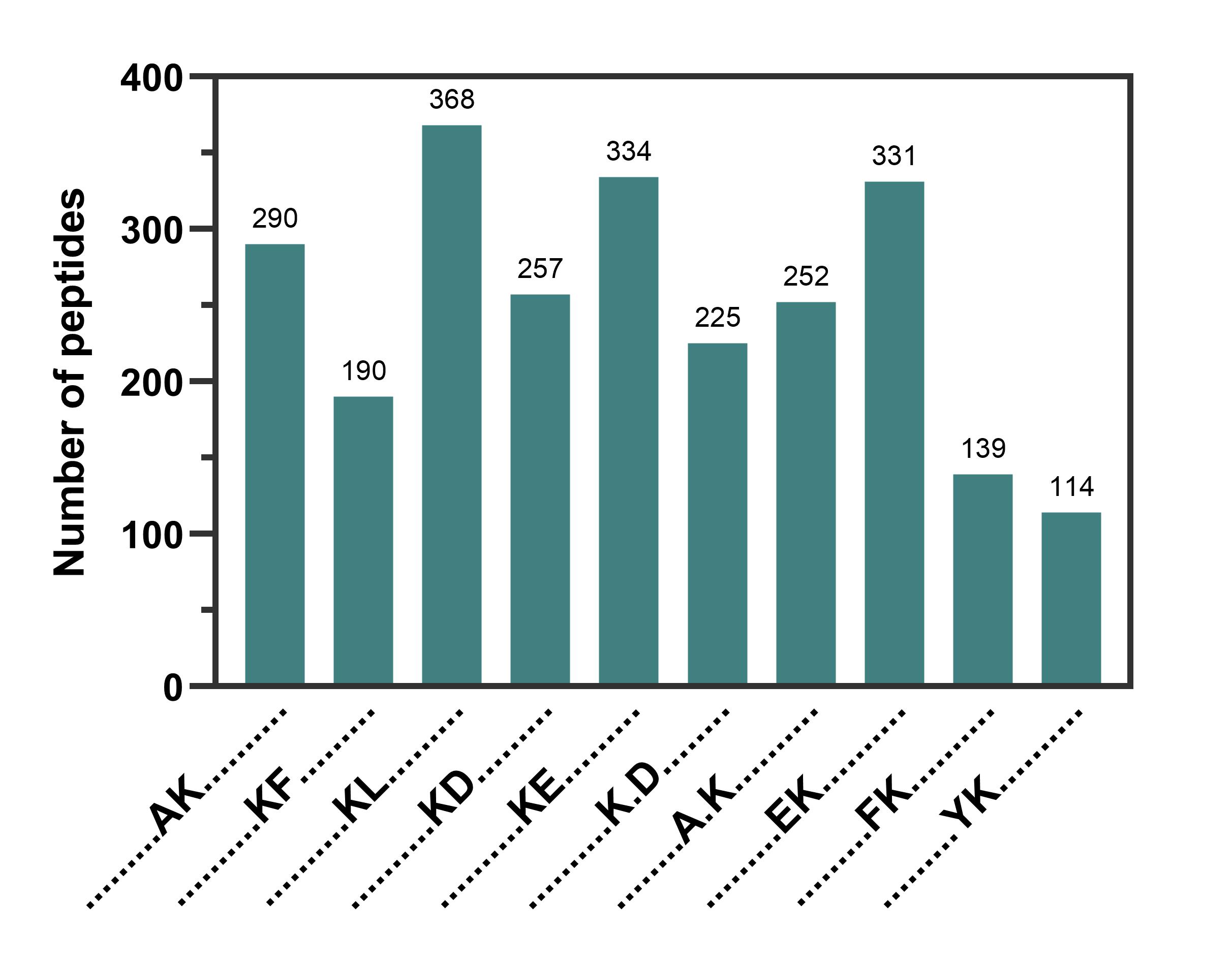


**Supplementary Figure 2.** Statistics of consensus motifs of polypeptides containing crotonylation modification sites. Kcr is the meaning of crotonylated lysine, K Lysine, A Alanine, F Phenylalanine, L leucine, D Aspartic, E glutamic acid, Y Tyrosine, respectively. “..........” represents any single amino acid.


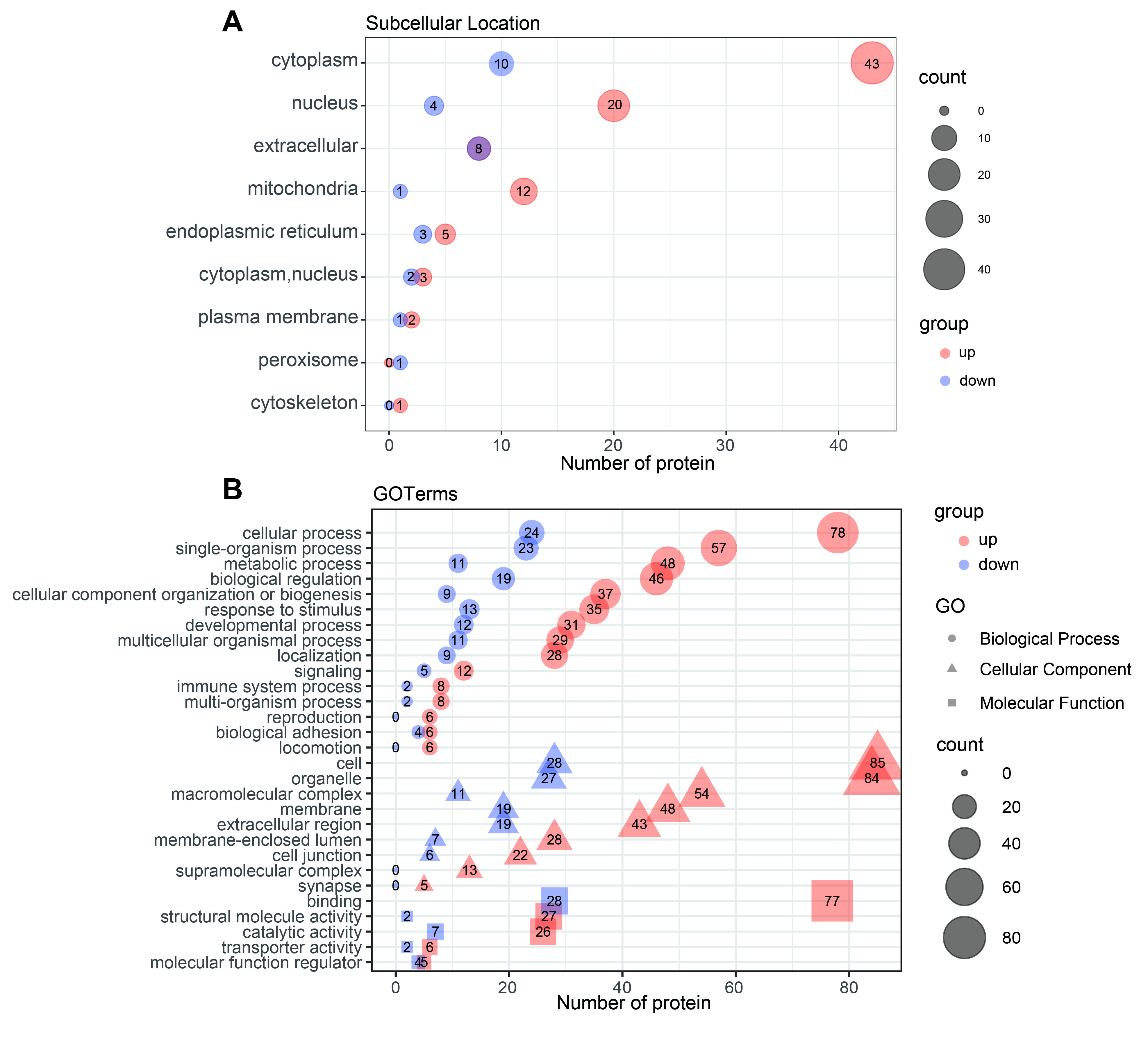


**Supplementary Figure 3.** Statistical analysis of the distribution of the quantified crotonylated protein in the GO secondary annotation, and classification statistics of the subcellular structure of differentially modified proteins in the comparison group. **(A)** Up-regulation and down-regulation of the location and distribution of the subcellular structure of the protein corresponding to the crotonylation site (PDGF vs Con). **(B)** Up-regulation and down-regulation of the distribution of crotonylation sites in GO secondary annotations (PDGF vs Con).


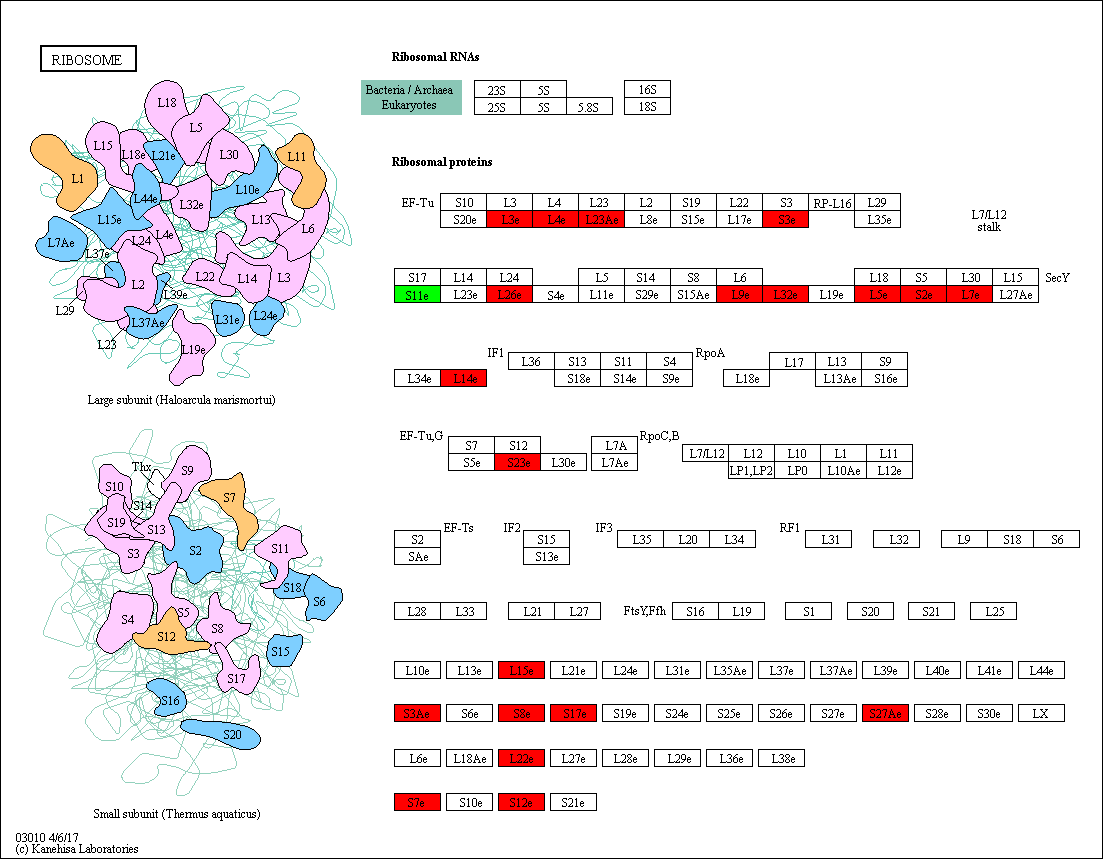


**Supplementary Figure 4.** Visual display of a certain KEGG pathway (PDGF vs Con) where the crotonylated protein corresponding to differentially modified sites is significantly enriched. The red in the figure shows the modification level up-regulated protein; the bright green shows the modification level down-regulated protein; and the yellow shows multiple proteins in this node, including proteins in up-regulated and down-regulated levels.


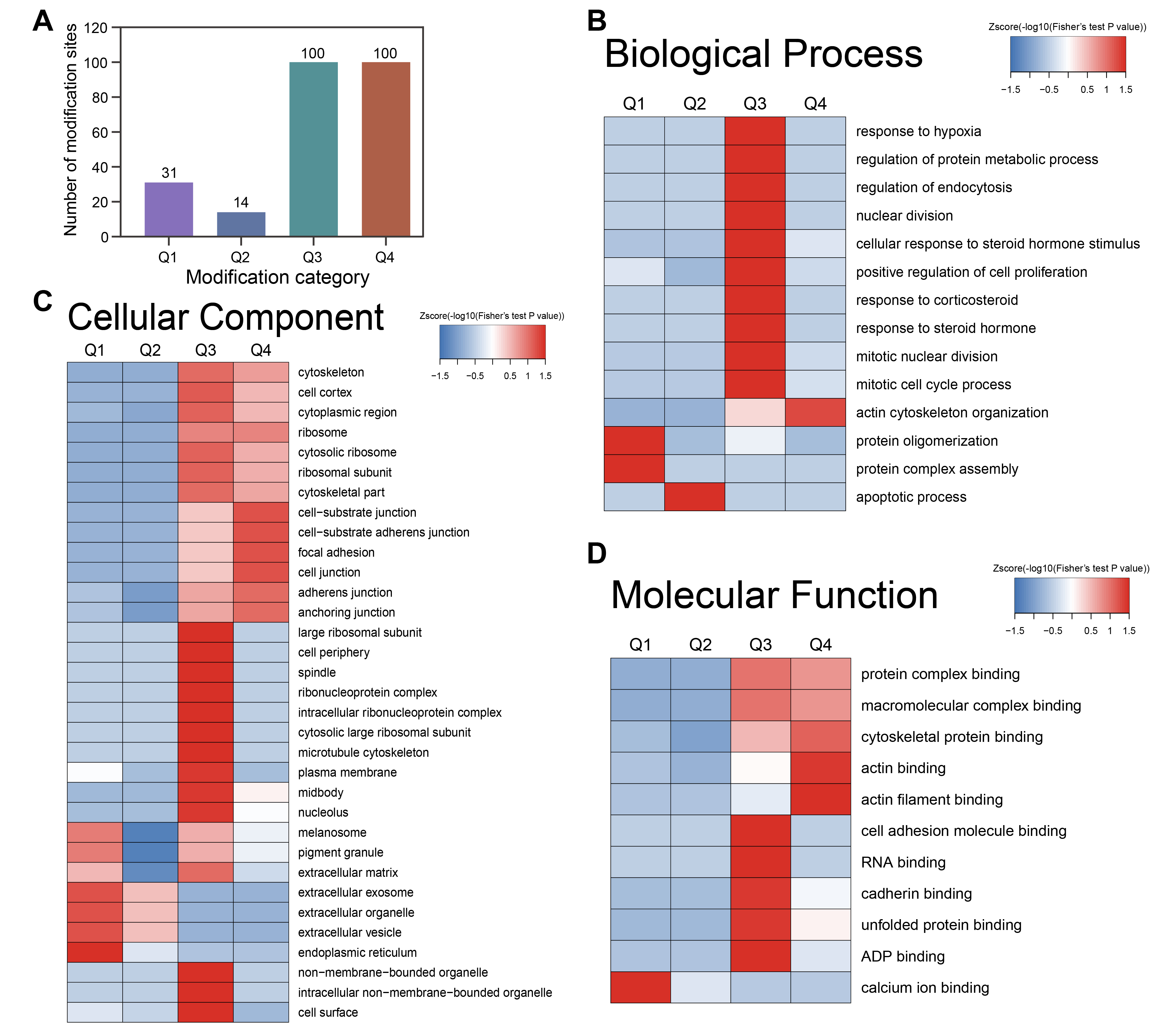


**Supplementary Figure 5.** Enrichment of GO and cluster analysis of crotonylated proteins with different modification levels in different differential expression folds. **(A)** Number distribution of differentially modified proteins which are divided into Q1-Q4 according to fold changes: Q1 (0< Ratio ≤ 1/1.5), Q2 (1/1.5 < Ratio ≤ 1/1.3), Q3 (1.3 < Ratio ≤1.5) and Q4 (Ratio >1.5) (Ratio represents fold changes of PDGF vs Con). Cluster analysis heat map based on GO enrichment. Three categories of GO including biological process **(B)**, cellular component **(C)** and molecular function **(D)**.


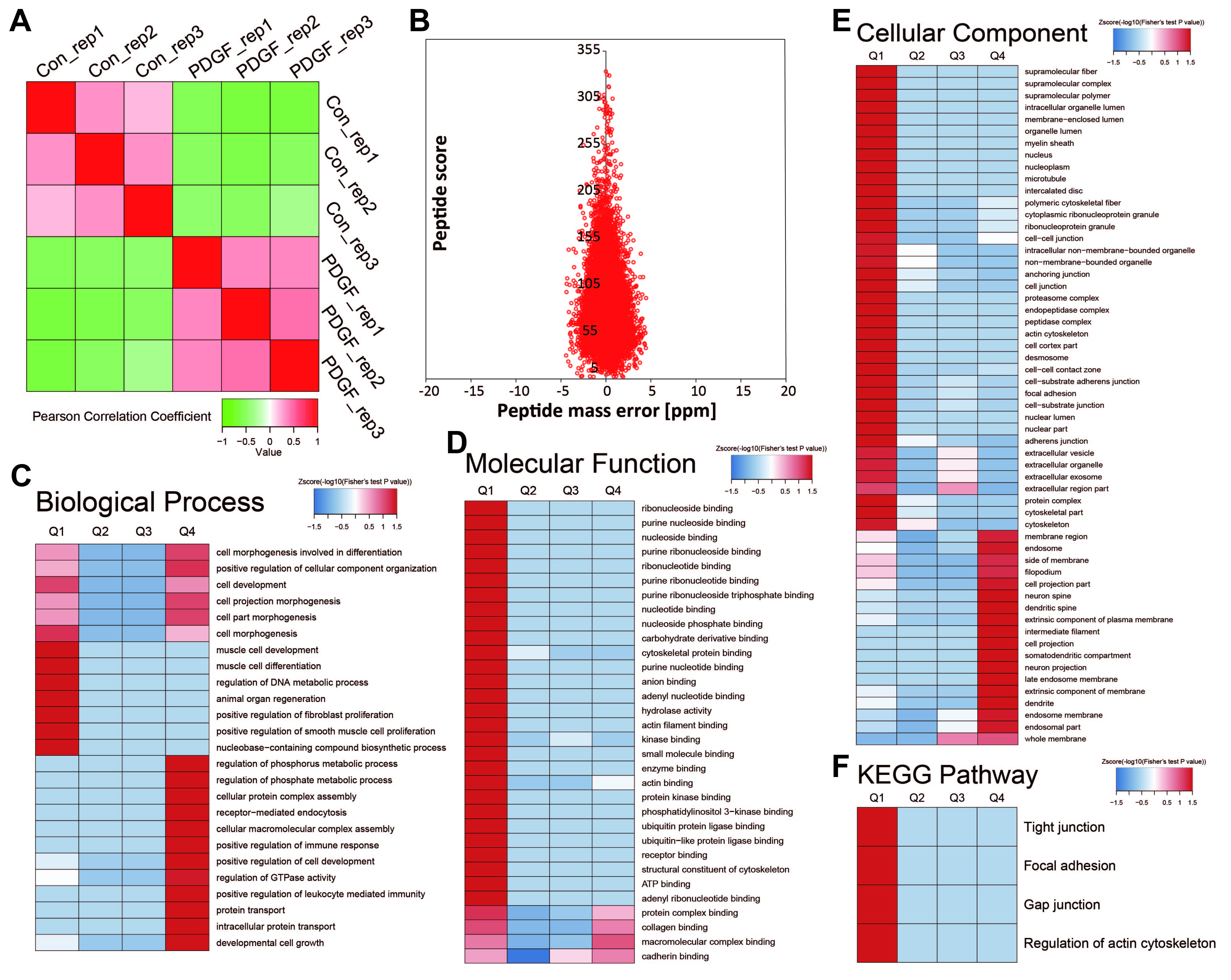


**Supplementary Figure 6.** Enrichment of GO and cluster analysis of ubiquitinated proteins with different modification levels in different differential expression folds. **(A)** Three biological replicates of VSMCs before or after PDGF-BB treatment. **(B)** MS identification. Functional enrichment-based clustering analysis for the quantified ubiquitylome on biological process analysis **(C)**, molecular function analysis **(D)**, cellular component analysis **(E)** and KEGG pathway **(F)**.


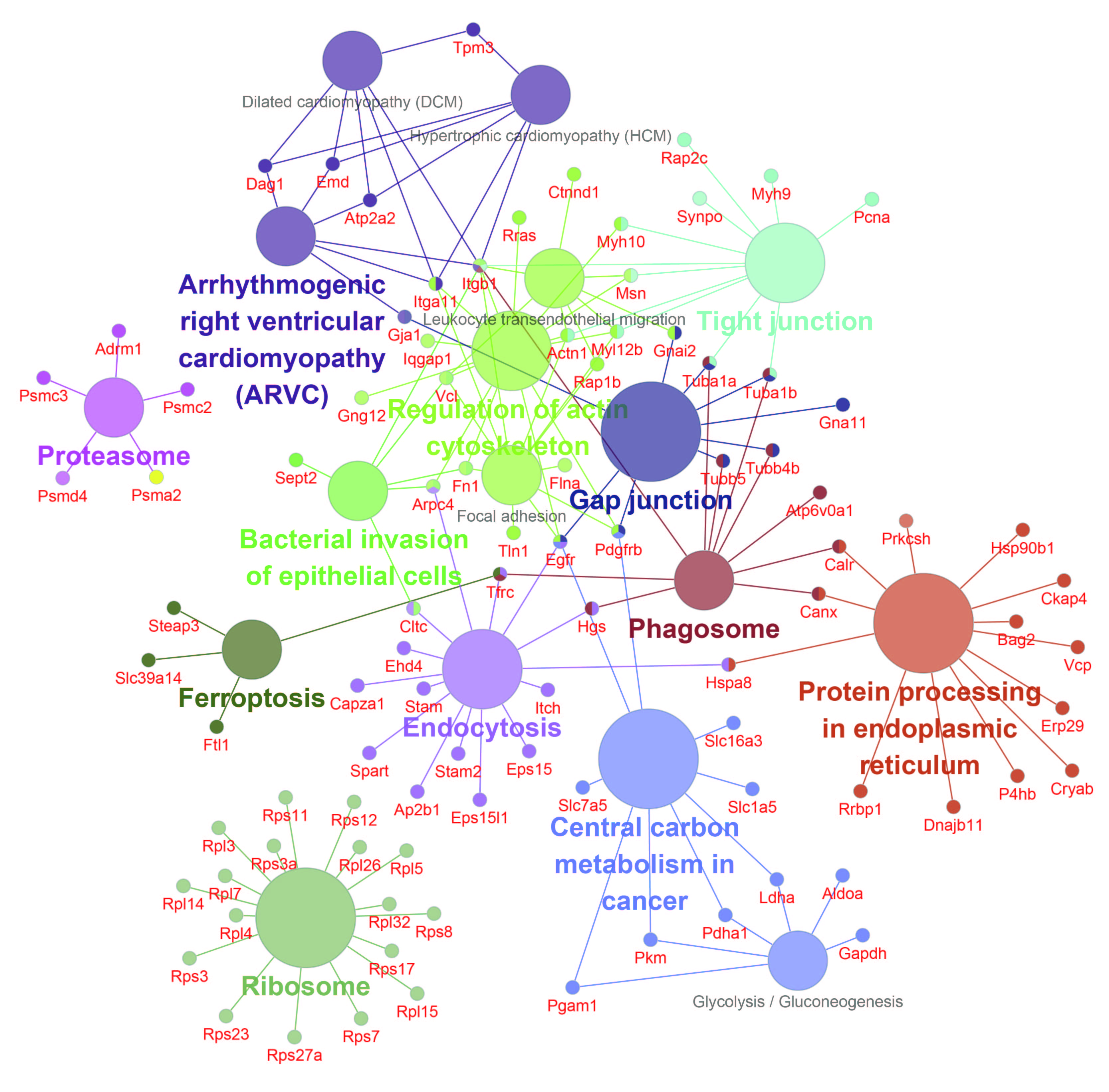


**Supplementary Figure 7.** Enrichment and cluster analysis of pathways network between crotonylation and ubiquitination.
